# Multi-omics exploration of chaperone-mediated immune-proteostasis crosstalk in vascular dementia and identification of diagnostic biomarkers

**DOI:** 10.3389/fimmu.2025.1615540

**Published:** 2025-07-30

**Authors:** Wentong Li, Yiyi Zhang, Chuanhong Li, Mingyang Jiang, Dong Wang, Luomeng Chao, Yuxia Yang

**Affiliations:** ^1^ College of Computer Science and Technology, Inner Mongolia MINZU University, Tongliao, China; ^2^ Department Oncology of Mongolian-Western Medicine, Affiliated Hospital of Inner Mongolia MINZU University, Tongliao, China; ^3^ College of Animal Science and Technology, Inner Mongolia MINZU University, Tongliao, China; ^4^ Inner Mongolia Rambo Testing Technology Limited Company, Tongliao, China; ^5^ Inner Mongolia Engineering Technology Research Center for Prevention and Control of Beef Cattle Diseases, Tongliao, China

**Keywords:** vascular dementia, diagnostic biomarkers, machine learning, single-cell transcriptome analysis, proteostasis crosstalk

## Abstract

**Introduction:**

Vascular dementia (VaD), the second most prevalent form of dementia globally, remains insufficiently understood in terms of its molecular mechanisms and diagnostic biomarkers. This study aims to elucidate the regulatory network and diagnostic potential of the molecular chaperone system in VaD through the integration of multi-omics data and machine learning algorithms.

**Methods:**

Transcriptomic data from frontal and temporal cortex (GSE122063, n=15)and white matter (GSE282111, n=8) samples were obtained from the GEO database. Differentially expressed genes (DEGs) were identified using the limma package (log2FC>0.656, *p<*0.05). Protein-protein interaction (PPI) networks were constructed using the STRING database. Biomarker validation was performed through cross-validation using LASSO, SVM-RFE, and Random Forest algorithms. Immune microenvironment analysis was conducted using CIBERSORT, while single-cell transcriptomics was analyzed within the Seurat framework.

**Results:**

A total of 897 DEGs were identified, with functional enrichment analysis revealing significant involvement in T cell activation (*p=*2.84×10^-3^), neuroactive ligand-receptor interaction (*p=*6.01×10^-4^), and osteoclast differentiation (NES=2.83). PPI network analysis identified HSP90AA1, HSPA1B, and DNAJB1 as core hub genes (degree centrality >20). Machine learning validation demonstrated their combined exceptional diagnostic efficacy (AUC=0.963, F1 = 0.88). Immune analysis revealed that this molecular chaperone axis modulates neuroinflammation by suppressing naive B cell differentiation (61% reduction) and activating Tregs (55.53% increase). Single-cell resolution analysis showed HSP90AA1 to be specifically overexpressed in oligodendrocytes (72.23%), significantly correlating with glial depletion (4.56% decrease in oligodendrocytes, *p<*0.01) and aberrant neuronal proliferation (144.23% increase, *p=*0.0032). *In vivo* experiments utilized a bilateral common carotid artery stenosis (BCAS) mouse model to simulate human vascular dementia (VaD), with further validation through Morris water maze testing. The BCAS group exhibited significantly upregulated mRNA expression of *HSP90AA1*, *HSPA1B*, and *DNAJB1*, consistent with integrated bioinformatics analysis results.

**Conclusion:**

This study elucidates the HSP90AA1-HSPA1B-DNAJB1 network as a key driver of VaD pathogenesis through dual mechanisms of protein homeostasis and immune reprogramming. The diagnostic performance of this network significantly surpasses traditional biomarkers (ΔAUC≥14.3%), offering novel targets for precision diagnostics and therapeutics. However, further validation with larger cohorts is necessary to assess its clinical translational potential.

## 1 Introduction

Vascular Dementia (VaD), second only to Alzheimer’s disease in prevalence among dementia types, is characterized primarily by cognitive impairment resulting from cerebrovascular pathology (1, 2). In the context of accelerating global aging, the incidence of VaD continues to rise. However, current diagnostic paradigms still rely heavily on neuropsychological scales and imaging assessments, lacking precise molecular biomarkers (3–5). Multi-omics synergistic modulation refers to the integrated analysis of biological data across multiple layers, including genomics, transcriptomics, and proteomics. This approach uncovers the interactions and cooperative effects among different molecular systems, enabling a more comprehensive understanding of disease mechanisms than any single omics layer alone. The recent revolutionary advancements in multi-omics technologies have provided novel perspectives for deciphering the molecular mechanisms underlying VaD. Transcriptomics has unveiled the activation patterns of neuroinflammatory and oxidative stress pathways, while proteomics has highlighted the crucial role of molecular chaperone systems in neuronal injury repair (6). Nevertheless, the heterogeneous pathological progression of VaD involves complex interactions within the neuro-immuno-vascular network, rendering single-omics data insufficient for a comprehensive molecular landscape delineation. Against this backdrop, the integration of multi-omics data with machine learning algorithms for systematic mining of core regulatory networks and diagnostic biomarkers in VaD has emerged as a frontier in neurodegenerative disease research. This approach holds the promise of unraveling the intricate molecular tapestry underlying VaD pathogenesis, potentially leading to more accurate diagnostic tools and targeted therapeutic strategies (7). By leveraging the synergistic power of diverse omics platforms and advanced computational methods, researchers aim to capture the multifaceted nature of VaD, encompassing transcriptional, proteomic, and metabolic alterations across various cell types and brain regions. The imperative for such integrative approaches is underscored by the limitations of current VaD management strategies, which are hampered by delayed diagnosis and the absence of disease-modifying treatments. By elucidating the molecular signatures and regulatory networks specific to VaD, this research paradigm aspires to identify novel biomarkers with enhanced diagnostic accuracy and prognostic value. Furthermore, the insights gained from these comprehensive analyses may illuminate potential therapeutic targets, paving the way for personalized interventions that address the complex pathophysiology of VaD more effectively (8–10).

Despite the documented neuroprotective role of Heat Shock Proteins (HSPs) in Alzheimer’s disease, their functional significance in Vascular Dementia (VaD) remains elusive (11, 12). Current limitations in this field are manifold: (i) Most studies focus on single-omics perspectives, lacking a systematic analysis across transcriptomics, protein-protein interaction networks, and the immune microenvironment; (ii) Traditional differential gene screening methods are susceptible to batch effects and struggle to distinguish driver genes from concomitant phenomena; (iii) Diagnostic biomarker studies often rely on single-model validation, lacking the robustness of multi-algorithm cross-validation; (iv) The mechanistic role of immune cell subpopulations in VaD remains controversial, particularly the dynamic relationship between regulatory T cells (Tregs) and B cell differentiation (13). These gaps, particularly the lack of integrative studies on immune-proteostasis interactions, significantly impede the development of precision diagnostic and therapeutic strategies for VaD.

This study focuses on the molecular chaperone-immune regulatory axis mediated by HSP90AA1, HSPA1B, and DNAJB1, proposing a core hypothesis that “protein homeostasis imbalance drives neuroinflammation in VaD.” Leveraging transcriptomic data from VaD patients’ brain tissue and peripheral blood single cells in the GEO database, we aim to elucidate the molecular mechanisms by which these genes influence neuroinflammatory processes through modulation of the Tregs/B cell balance. This investigation integrates differential expression profiles, protein-protein interaction network topological analysis, and machine learning-driven feature selection. Our approach offers a novel perspective on immune-metabolic reprogramming in VaD and lays the theoretical foundation for developing HSP-targeted neuroprotective therapies.

Key questions addressed in this study include: (i) How do VaD-characteristic differentially expressed genes regulate the immune microenvironment through the molecular chaperone system? (ii) Does the HSP90AA1*-*HSPA1B*-*DNAJB1 axis possess biological plausibility as a cross-omics diagnostic biomarker? (iii) At single-cell resolution, how does the cell type-specific expression pattern of these genes influence neuroglia homeostasis? Research objectives encompass constructing a multi-omics regulatory network for VaD, validating the diagnostic efficacy of core genes, and elucidating their immune regulatory mechanisms. Resolving these questions will fill critical gaps in our understanding of the molecular chaperone system’s function in VaD and provide new targets for stratified diagnosis and treatment.

Existing evidence suggests that HSP90AA1 promotes angiogenesis following ischemic brain injury by stabilizing HIF-1α (14), while HSPA1B overexpression inhibits abnormal Tau protein phosphorylation (15). In terms of immune regulation, DNAJB1 has been shown to exacerbate neuroinflammation through activation of the TLR4/NF-κB pathway. However, these studies are largely confined to animal models or *in vitro* experiments, lacking systematic validation at the human tissue level. Recent single-cell sequencing studies have revealed a negative correlation between microglial HSPs expression and cognitive decline (16), but have yet to establish dynamic connections with peripheral immune cells. Our study innovatively combines the molecular chaperone system with immune infiltration analysis, transcending the limitations of the “neuro-immune” binary paradigm prevalent in existing literature.

We employ a stepwise analytical strategy: (i) Curating and separately analyzing the GSE122063 (frontal and temporal cortex) and GSE282111 (white matter) datasets using the limma package to identify robust differential genes; (ii) Constructing high-confidence STRING interaction networks to identify hub genes through degree centrality and betweenness centrality; (iii) Cross-validating biomarkers using LASSO, SVM-RFE, and Random Forest algorithms; (iv) Analyzing immune cell heterogeneity through CIBERSORT deconvolution and single-cell transcriptomics. Methodological advantages include employing quantile normalization to eliminate inter-platform variations, assessing multi-model feature overlap through Jaccard coefficients, and enhancing biomarker reliability through a dual validation system combining ROC curves and confusion matrices.

## 2 Methods

### 2.1 Multi-omics data acquisition and preprocessing

This study systematically curated Vascular Dementia (VaD)-related transcriptomic datasets from the Gene Expression Omnibus (GEO) database. Inclusion criteria encompassed: (i) GSE122063 dataset (11 healthy controls and 4 VaD patient frontal and temporal cortex samples) based on the Illumina HiSeq 2500 platform (GPL16699);(ii) GSE282111 dataset (4 healthy controls and 4 VaD patient white matter samples) generated using the NovaSeq 6000 platform (GPL24676); (iii) GSE186798 dataset, comprising 30 healthy controls and 30 VaD patient frontal cortex samples, profiled using the Affymetrix Clariom S Human array (GPL23159). Raw CEL files underwent standardized parsing via the GEOquery package, with subsequent elimination of low-quality probes and batch effect-confounded data (17, 18).

### 2.2 Systematic identification of differentially expressed genes

A multi-stage differential analysis was implemented using the R/Bioconductor computational framework (v4.4.2). Initially, stringent quality control was applied to the raw expression matrix, excluding gene rows with >5% missing expression values and probes lacking annotation information, while retaining unique gene identifiers corresponding to maximum expression values (19). To mitigate technical variability across different microarray and sequencing platforms, batch effect correction was applied using the ComBat algorithm from the sva package—an empirical Bayes-based method extensively validated for removing non-biological variance in transcriptomic data (20). The expression matrices were then subjected to quantile normalization and log2 transformation to reduce technical noise and approximate a Gaussian distribution (**Supplementary Figure S1**).

Differentially Expressed Genes (DEGs) were identified using the limma package (v3.58.1) to construct linear models, with significance thresholds set at adjusted *p<*0.05 and log2FoldChange>0.656 (corresponding to a 1.6-fold expression difference) (21). Hierarchical clustering heatmaps were generated using the ComplexHeatmap package, while volcano plots illustrating the significance-effect size distribution of differential genes were created using the ggplot2 package (22).

### 2.3 Construction of multi-dimensional functional annotation system

To systematically decipher the biological significance of DEGs, an integrated enrichment analysis strategy was adopted. The clusterProfiler package (v4.10.0) was utilized to perform three-tier Gene Ontology (GO) annotation, encompassing Biological Process (BP), Molecular Function (MF), and Cellular Component (CC) dimensions (23) (**Supplementary Tables 1–3**). Significant signal transduction networks (*FDR*<0.05) were identified by mining the Kyoto Encyclopedia of Genes and Genomes (KEGG) pathway database based on a hypergeometric distribution model (24, 25). Furthermore, a global Gene Set Enrichment Analysis (GSEA) strategy was implemented using predefined gene sets from the Molecular Signatures Database (MSigDB v7.5.1) to quantify the synergistic regulatory intensity of functional modules through a weighted enrichment scoring algorithm (26).

### 2.4 Protein interaction network topological analysis

High-confidence protein-protein interaction networks (combined score ≥0.700) were constructed based on the STRING database (v11.5), with network visualization and topological parameter calculations performed using the Cytoscape platform (v3.8.2) (27–29). Key hub proteins were identified using the Molecular Complex Detection (MCODE) algorithm, with parameters set as follows: degree cutoff = 2, score cutoff = 0.2, k-core value = 2, and max depth = 100, to screen for topologically dense functional modules (30–32). Concurrently, core regulatory factors were cross-validated by integrating Degree Centrality and Betweenness Centrality metrics (33–35).

### 2.5 Machine learning-based selection of common feature genes

This study employed three widely-used machine learning algorithms to identify potential biomarkers for VaD. During model development, an imbalance in sample sizes between VaD and healthy controls was noted. To mitigate bias from this class imbalance, model performance was evaluated using stratified cross-validation and out-of-bag (OOB) error estimation, ensuring balanced representation across training and validation subsets. Firstly, the Least Absolute Shrinkage and Selection Operator (LASSO) regression model was applied. A five-fold cross-validation procedure was used to optimize the regularization parameter λ by minimizing the mean prediction error. The final model, retrained on the entire dataset with the optimal λ, yielded a subset of non-zero coefficient features as initial candidate genes. Leveraging L1 regularization, LASSO enables embedded feature selection while constraining model complexity. When combined with cross-validation, this approach effectively reduces overfitting and enhances generalizability. It offers strong stability and robustness in the context of high-dimensional, small-sample omics data, thereby reinforcing the reliability of the selected biomarkers (36, 37). The optimization objective function for LASSO regression is defined as:


$${\text{β}}^{\text{LASSO}}=\text{ar}{\text{g}}_{\text{β}}\text{min}\{\sum_{\text{i}=1}^{\text{n}}{\left({\text{y}}_{\text{i}}-{\text{β}}_{0}-\sum_{\text{j}=1}^{\text{p}}{\text{x}}_{\text{ij}}{\text{β}}_{\text{j}}\right)}^{2}+\lambda \sum_{\text{j}=1}^{\text{p}}|{\text{β}}_{\text{j}}|\}$$

Where $\sum_{i=1}^{n}{\left(y_{i}-\beta_{0}-\sum_{j=1}^{p}x_{ij}\beta_{j}\right)}^{2}$ represents the residual sum of squares, $\lambda \sum_{j=1}^{p}\left|\beta_{j}\right|$ is the L1 regularization term, *λ* denotes the regularization parameter, facilitating the selection of genes with the highest predictive value through the introduction of L1 regularization.

Secondly, the Support Vector Machine - Recursive Feature Elimination (SVM-RFE) method was utilized. A five-fold cross-validation framework was applied for both model training and feature selection, using four folds for training and one for validation in each iteration to minimize information leakage and reduce overfitting risk. In each iteration, the feature with the lowest ranking coefficient is eliminated, ultimately yielding a descending order of all feature attributes (38). The feature rankings from each fold were then aggregated to derive an averaged consensus order. To further enhance model robustness and prevent redundancy-related overfitting, classification accuracy and error rates were evaluated across varying numbers of top-ranked features, enabling the selection of an optimal feature subset.

Lastly, the Random Forest (RF) algorithm, an ensemble learning method based on decision trees, was employed. Model performance was evaluated using the Out-of-Bag (OOB) estimation strategy, wherein each tree is trained on a bootstrapped subset (63% of the data), and validated on the remaining (37%) unseen samples. This ensemble voting mechanism effectively reduces variance, enhances generalizability, and mitigates overfitting, while simultaneously enabling the assessment of feature importance (39–41). The feature importance is calculated as follows:


$$\text{MeanDecreaseGin}{\text{i}}_{\text{j}}=\sum_{\text{t}=1}^{\text{T}}\sum_{\text{n}\in N_{j}^{t}}△\text{Gin}i_{j}^{t}(\text{n})$$

Where *T* is the total number of decision trees, $N_{j}^{t}$ represents the node set split by feature in the *t* tree. Genes consistently identified by both LASSO and RF algorithms were prioritized as candidate biomarkers, ensuring methodological robustness and biological relevance.

By integrating the results from LASSO, SVM-RFE, and RF, feature genes commonly identified by all three methods were selected as candidate biomarkers, thereby enhancing the reliability and robustness of the screening process (42).

### 2.6 Multi-dimensional validation of diagnostic efficacy

This study established a multi-dimensional diagnostic efficacy evaluation system, implementing Receiver Operating Characteristic (ROC) curve analysis using the pROC package (v1.18.5) in R. Through a continuous threshold scanning strategy, the classification performance of the feature gene set was systematically quantified: constructing a parametric space trajectory with sensitivity [TP/(TP+FN)] as the ordinate and 1-specificity [FP/(FP+TN)] as the abscissa (43, 44). The Area Under Curve (AUC), serving as a non-parametric statistic, was calculated based on the Mann-Whitney U test principle. The DeLong algorithm was employed to compute 95% confidence intervals (45). An AUC value approaching 1 indicates perfect discrimination capability of the feature gene set; AUC>0.9 denotes excellent diagnostic efficacy, 0.8-0.9 good, 0.7-0.8 moderate, and <0.7 suggests limited clinical applicability.

Concurrently, the study utilized the caret package to construct confusion matrices, calculating metrics such as accuracy, recall, and F1 score. An F1 score approaching 1 represents higher accuracy and recall of the model, with these two values converging numerically (46). In sample classification, this implies a more perfect overall classification effect, with all predicted results closely approximating true values, indicating enhanced reliability and effectiveness of the model in identifying and distinguishing sample categories.

To further assess gene performance in classification tasks, visualization was conducted using the ggplot2 and ggpubr packages, facilitating a more intuitive and comprehensive analysis of the genes’ diagnostic efficacy (47–49).

### 2.7 Immune infiltration analysis

The CIBERSORT algorithm was employed to quantitatively assess the immune cell composition within samples. This algorithm, based on support vector regression principles, utilizes deconvolution analysis to parse mixed gene expression data into relative proportions of specific immune cell subsets (**Supplementary Table 4**). The CIBERSORT analysis was implemented using R, with boxplots generated to visualize differences in immune cell infiltration among different sample groups (50).

To explore potential associations between feature genes and the immune microenvironment, Pearson correlation analysis was conducted. The correlation coefficient r between feature gene expression levels and the abundance of various immune cell subsets was calculated using the following formula:


$$\text{γ}=\frac{\sum_{\text{i}=1}^{\text{n}}({\text{x}}_{\text{i}}-\bar{\text{x}})({\text{y}}_{\text{i}}-\bar{\text{y}})}{\sqrt{\sum_{\text{i}=1}^{\text{n}}{\left({\text{x}}_{\text{i}}-\bar{\text{x}}\right)}^{2}}\sqrt{\sum_{\text{i}=1}^{\text{n}}{\left({\text{y}}_{\text{i}}-\bar{\text{y}}\right)}^{2}}}$$

Where X and Y represent gene expression values and immune cell abundances, respectively, and $\bar{X}$ and $\bar{Y}$ denote their respective means. A threshold of *P* < 0.05 was set for statistical significance, elucidating potential regulatory relationships between feature genes and immune infiltration atterns (51).

### 2.8 Single-cell transcriptome analysis pipeline

This study systematically analyzed the single-cell transcriptome landscape of peripheral blood mononuclear cells (PBMCs) in vascular dementia (VaD) using the GSE282111 dataset, encompassing 4 VaD patients and 4 healthy controls. A multi-stage data processing approach was implemented based on the Seurat framework (v5.0.1): Initially, the LogNormalize algorithm was applied to preprocess and normalize the raw UMI count matrix (**Supplementary Figure S2**). Subsequently, the IntegrateLayers function was employed to execute a cross-sample integration strategy based on Canonical Correlation Analysis (CCA), mitigating technical variations between batches (**Supplementary Figure S3**). The PercentageFeatureSet function was utilized to quantify the proportion of mitochondrial gene expression (percent.mt), with stringent selection of high-quality cells (percent.mt ≤ 5%) for downstream analysis (52) (**Supplementary Figure S4**). Highly Variable Genes (HVGs) were identified using the FindVariableFeatures function, employing a variance stabilizing transformation (vst) strategy to select the top 2000 HVGs, capturing the primary sources of cellular heterogeneity (**Supplementary Figure S5**). Principal Component Analysis (PCA) was performed on the HVG matrix, extracting the first 50 principal components, with dimensional significance validated using the JackStraw algorithm. A shared nearest neighbor graph was constructed using the FindNeighbors function (k=20), followed by unsupervised cell clustering using the Louvain algorithm (resolution=0.8). Finally, the FindAllMarkers function (min.pct=0.25, logfc.threshold=0.25) was employed to identify differentially expressed markers for each cell cluster, with cell type annotation performed in reference to the Cell Marker database (v2.0) (53).

### 2.9 Experimental animals

Male C57BL/6 mice (8–10 weeks old, 22–25 g body weight) were obtained from SpeiPharm Biotechnology Co., Ltd. (Beijing, China) and maintained under specific pathogen-free (SPF) conditions in the Animal Science Laboratory of Inner Mongolia University for Nationalities with ad libitum access to food and water under a 12-h light/dark cycle. The bilateral common carotid artery stenosis (BCAS) mouse model was established as previously described (54), wherein microcoils (0.18 mm internal diameter) were surgically implanted to induce stenosis in both common carotid arteries (CCAs). Animals were randomly assigned using a random number table to either the BCAS group (vascular dementia model, VAD) or the control group, with six mice per group.

### 2.10 Morris water maze test

Cognitive function was assessed using the Morris water maze (MWM) paradigm, performed in accordance with established protocols (55, 56). The apparatus consisted of a circular pool (maintained at 24 ± 2°C) divided into four quadrants, with a 10-cm diameter escape platform submerged 1 cm below the water surface in the center of the southwest (target) quadrant. Following a 30-s habituation period, mice underwent a 6-day testing protocol comprising: (i) 4 days of directional navigation training, (ii) 1 day of navigation testing, and (iii) 1 day of spatial exploration test. All sessions were conducted at consistent diurnal timepoints. During acquisition, each mouse performed four consecutive trials daily from randomized starting quadrants (inter-trial interval: 60 min), with the platform positioned in quadrant IV. Trials were automatically terminated upon platform localization (dwell time >5 s) or after 60 s (guided to platform with 15-s rest). Navigation testing consisted of single 60-s trials initiated from quadrant II (maximal distance from target), with recording of: (i) escape latency, (ii) path length, and (iii) swimming velocity. The probe test (120-s duration) evaluated spatial memory retention by quantifying: (i) target quadrant occupancy time, and (ii) platform location crossings, following platform removal. All behavioral parameters were quantified using automated video-tracking software (EthoVision, Noldus, Netherlands).

### 2.11 Quantitative real-time PCR analysis

Gene expression profiles of *HSP90AA1*, *HSPA1B* and *DNAJB1* in whole blood specimens were quantitatively assessed through reverse transcription-quantitative polymerase chain reaction (RT-qPCR) methodology. Total RNA isolation was achieved using TRIzol reagent (Invitrogen), with subsequent cDNA synthesis performed employing the RevertAid First Strand cDNA Synthesis Kit (Yeasen Biotechnology) in strict adherence to the manufacturer’s protocol. Amplification reactions were conducted utilizing SYBR Green Real-Time PCR Master Mix (Yeasen Biotechnology) on a StepOne Plus Real-Time PCR System (Applied Biosystems), with glyceraldehyde-3-phosphate dehydrogenase (GAPDH) serving as the endogenous control for data normalization. All qPCR assays were performed in technical triplicate across six biologically independent RNA preparations, with primer sequences detailed in **Table 1**.

**Table 1:** Primers used for qRT-PCR.

Genes	Forward sequences (5′-3′)	Reverse sequences (5′-3′)	Size
*HSP90AA1*	CCTGACGGACCCCAGTAAAC	TCCACAATGGTCAGGGTTCG	90
*HSPA1B*	GCACTGTACCAGGGGATTATG	TTCCCAGGCTACTGGAACACT	95
*DNAJB1*	CTCCTTCACCCTCTGATCCGC	CCATTAGCACCACCACTGCTT	147
*GAPDH*	CGGTGCTGAGTATGTCGTGGAGTC	GGCGGAGATGATGACCCTTTTG	100

## 3 Results

### 3.1 Transcriptome differential expression profile analysis

**Figure 1:**
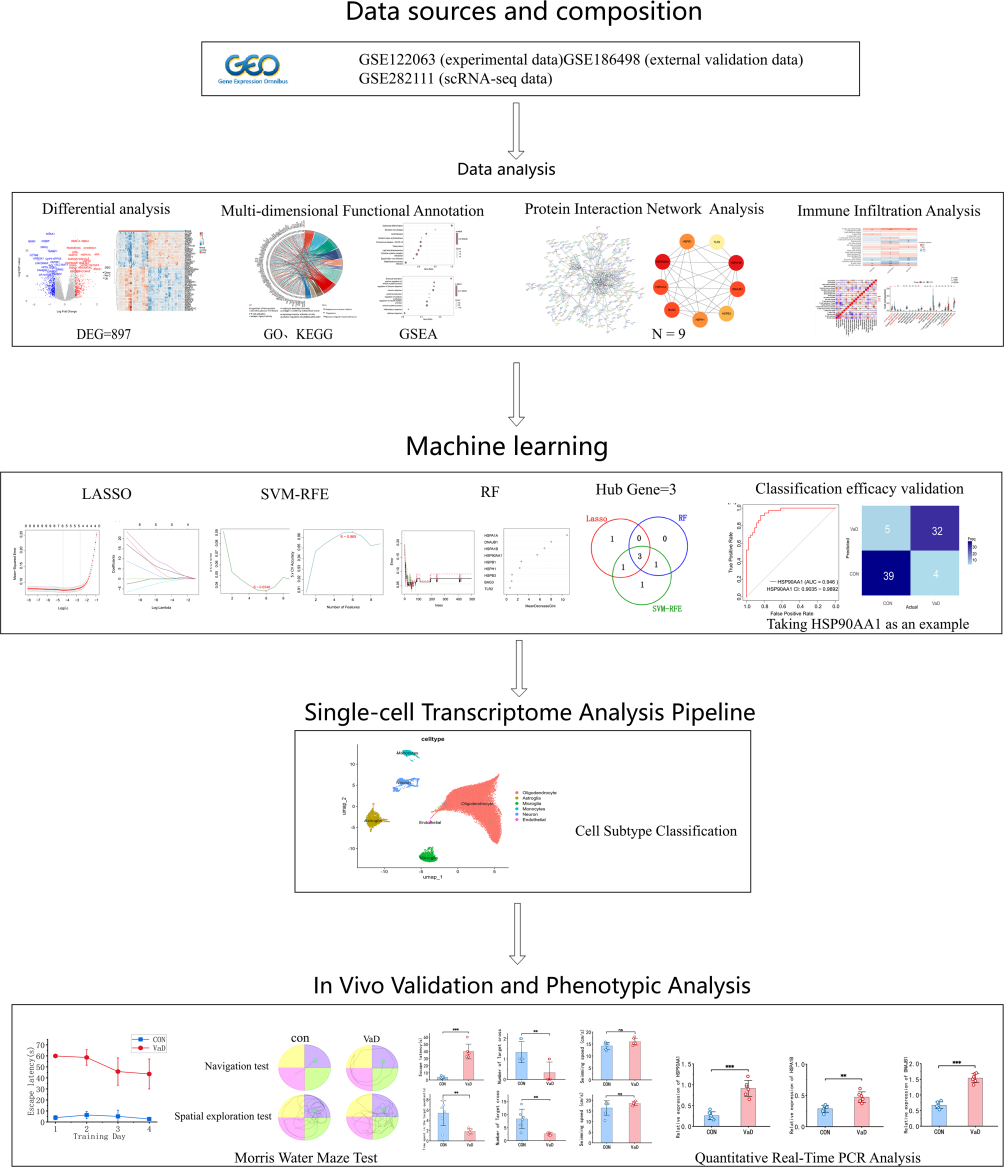
Schematic workflow of the study.

**Figure 2:**
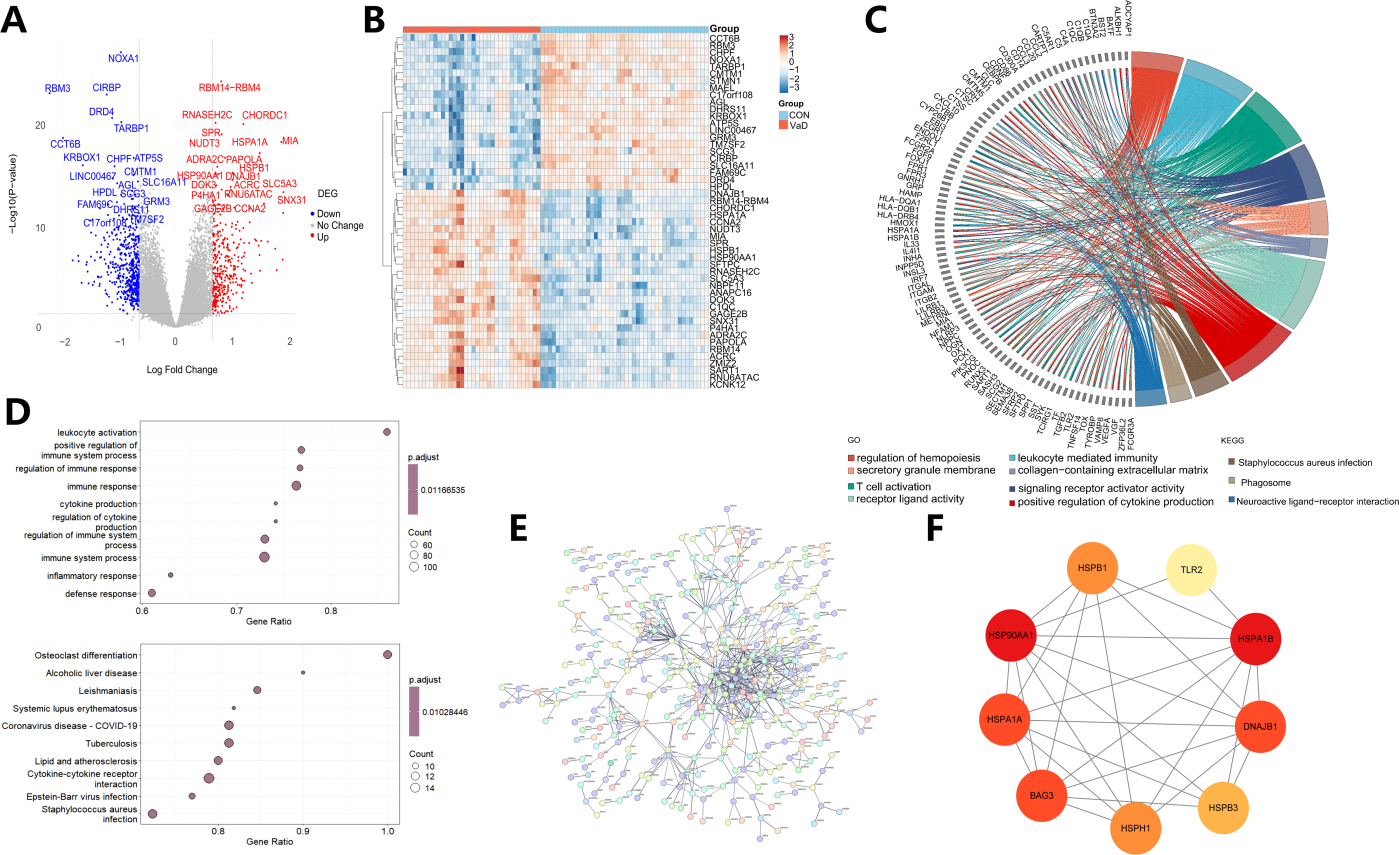
Multi-omics characterization of vascular dementia: differential gene expression signatures, functional network regulation, and hub protein identification. **(A)** Volcano plot illustrating the transcriptional landscape of vascular dementia, highlighting the top 20 most significantly dysregulated genes. Upregulated genes are depicted in red, while downregulated genes are color-coded in blue. Genes lacking statistically significant differential expression are shown in gray. **(B)** Hierarchical clustering analysis of the top 50 differentially expressed genes, revealing distinct transcriptional patterns across behavioral-associated samples. Each row corresponds to a gene, and each column represents an individual sample. Expression levels are visualized using a color gradient, with warmer hues (red) indicating higher expression and cooler tones (blue) denoting lower expression. **(C)** Gene Ontology (GO) enrichment analysis of differentially expressed genes and Kyoto Encyclopedia of Genes and Genomes (KEGG) pathway enrichment map. **(D)** GSEA-driven Gene Ontology analysis identifying the top 10 biologically coherent processes exhibiting concerted transcriptional dysregulation. Circular markers represent individual processes, with radii scaled to reflect participating gene set cardinality. GSEA-KEGG enrichment analysis plot. It shows the enrichment of the top 10 most significant pathways, with bubble size reflecting the number of enriched genes in each pathway. **(E)** Consensus protein-protein interaction (PPI) network reconstructed from multi-omics evidence streams (STRING v11.5; combined score > 0.7), depicting co-regulatory gene modules implicated in vascular dementia pathogenesis. Nodes represent gene products sized by degree centrality (DC ≥ 15), while edges encode experimentally validated interactions weighted by confidence metrics. **(F)** Molecular Complex Detection (MCODE)-derived topology refinement identifies a 9-gene topological hub orchestrating intermodular signaling dynamics. Chromatic encoding employs a continuous gradient spectrum (pale yellow → burnt sienna → cardinal red) proportional to eigenvector centrality metrics, where saturation intensity directly correlates with functional coherence scores.

**Figure 1** outlines the complete workflow. This study employed multidimensional bioinformatics analysis methods to systematically identify the characteristic transcriptome differential expression profile of vascular dementia (VaD). Following standardized data processing and statistical screening, 897 differentially expressed genes (DEGs) with significant biological relevance were ultimately identified, comprising 356 upregulated and 541 downregulated genes. A visualization analysis platform, constructed based on the R language ecosystem, generated a hierarchical clustering heatmap of the top 50 DEGs using the pheatmap package (**Figure 2B**), complemented by a volcano plot created with the ggplot2 package (**Figure 2A**).

### 3.2 Multidimensional functional annotation and pathway network

This study systematically integrated multi-level functional enrichment analysis strategies to annotate the biological functions of the 897 identified DEGs. Gene Ontology (GO) three-dimensional classification system analysis (**Figure 2C**) revealed that in the Biological Process (BP) dimension, DEGs were significantly enriched in key processes such as T lymphocyte activation regulation (*p*.adjust=2.84×10^-3^), positive regulation of cytokine generation (*p*.adjust=4.09×10^-3^), leukocyte-mediated immune effector processes (*p*.adjust=2.71×10^-3^), and hematopoietic lineage differentiation (*p*.adjust=2.71×10^-3^). At the Cellular Component (CC) level, DEGs were specifically localized to collagen matrix (p.adjust=3.01×10^-2^) and secretory granule membrane structures (*p*.adjust=3.01×10^-2^). Molecular Function (MF) analysis unveiled their significant involvement in signal receptor activation (*p*.adjust=1.62×10^-2^) and ligand-receptor interactions (*p*.adjust=1.62×10^-2^). These data suggest that differential genes may participate in VaD pathological processes by regulating innate immune responses, transmembrane signal transduction, and hematopoietic stem cell-directed differentiation.

KEGG pathway enrichment analysis further elucidated the pivotal roles of these genes in molecular interaction networks, with significant enrichment in pathways such as neuroactive ligand-receptor interaction (EF≈2.58, *p*.adjust=6.01×10^-4^), phagosome maturation (EF≈3.57, *p*.adjust=2.76×10^-5^), and Staphylococcus aureus infection (EF≈4.55, *p*.adjust=1.74×10^-5^). Notably, systematic exploration based on Gene Set Enrichment Analysis (GSEA) (**Figure 2D**) revealed significant clustering tendencies in GO functional sets related to immune pathways, including leukocyte activation (NES=3.84, *p*.adjust=1.34×10^-2^) and positive regulation of immune system processes (NES=4.20, *p*.adjust=1.34×10^-2^). The KEGG pathway set highlighted coordinated regulation of pathological processes such as osteoclast differentiation (NES=2.83, *p*.adjust=8.07×10^-3^), alcoholic liver disease (NES=2.59, *p*.adjust=8.07×10^-3^), and tuberculosis infection (NES=2.70, *p*.adjust=8.07×10^-3^). This multi-level enrichment profile suggests that the onset and progression of VaD may be closely associated with cross-system mechanisms including neuro-immune axis imbalance, aberrant pathogen pattern recognition, and bone metabolism homeostasis dysregulation.

### 3.3 Identification of core modules in protein-protein interaction network

This study systematically constructed a genome-wide protein-protein interaction (PPI) network, employing multidimensional association predictions for the 897 common differentially expressed genes based on the STRING database (v11.5). Network topological feature analysis was performed using Cytoscape (v3.8.2) (**Figure 2E**). Through combined screening of topological parameters including Betweenness, Degree, and Closeness, nine core regulatory genes were successfully identified: HSP90AA1 (Degree=22), HSPA1A (Degree=7), BAG3 (Degree=9), HSPH1 (Degree=6), HSPB3 (Degree=6), DNAJB1 (Degree=10), HSPA1B (Degree=9), *TLR2* (Degree=24), and HSPB1 (Degree=7). Their interaction module exhibited significant small-world network characteristics (clustering coefficient=0.608, average path length=4.23).

HSP90AA1 and HSPA1B emerged as network hub nodes (Betweenness centrality > 0.01), mediating the ubiquitination and degradation of misfolded proteins through dynamic regulation of the assembly-disassembly equilibrium of molecular chaperone complexes. Experimental evidence indicates that HSP90AA1 (Heat Shock Protein 90α subtype), an ATP-dependent molecular chaperone, participates in the cascade reactions of ischemia-reperfusion injury by modulating conformational changes of transcription factors such as NF-κB and HIF-1α. Conversely, transcriptional upregulation of HSPA1B (an HSP70 family member) can effectively eliminate abnormal protein aggregates induced by oxidative stress through enhancement of the HSP70-proteasome axis function, thereby maintaining neuronal mitochondrial homeostasis (**Figure 2F**). The topological advantage of this interaction module suggests that dysregulation of the molecular chaperone-mediated protein quality control system may be a core molecular mechanism underlying the neurodegenerative pathology in VaD.

### 3.4 Machine learning-driven biomarker screening

**Figure 3:**
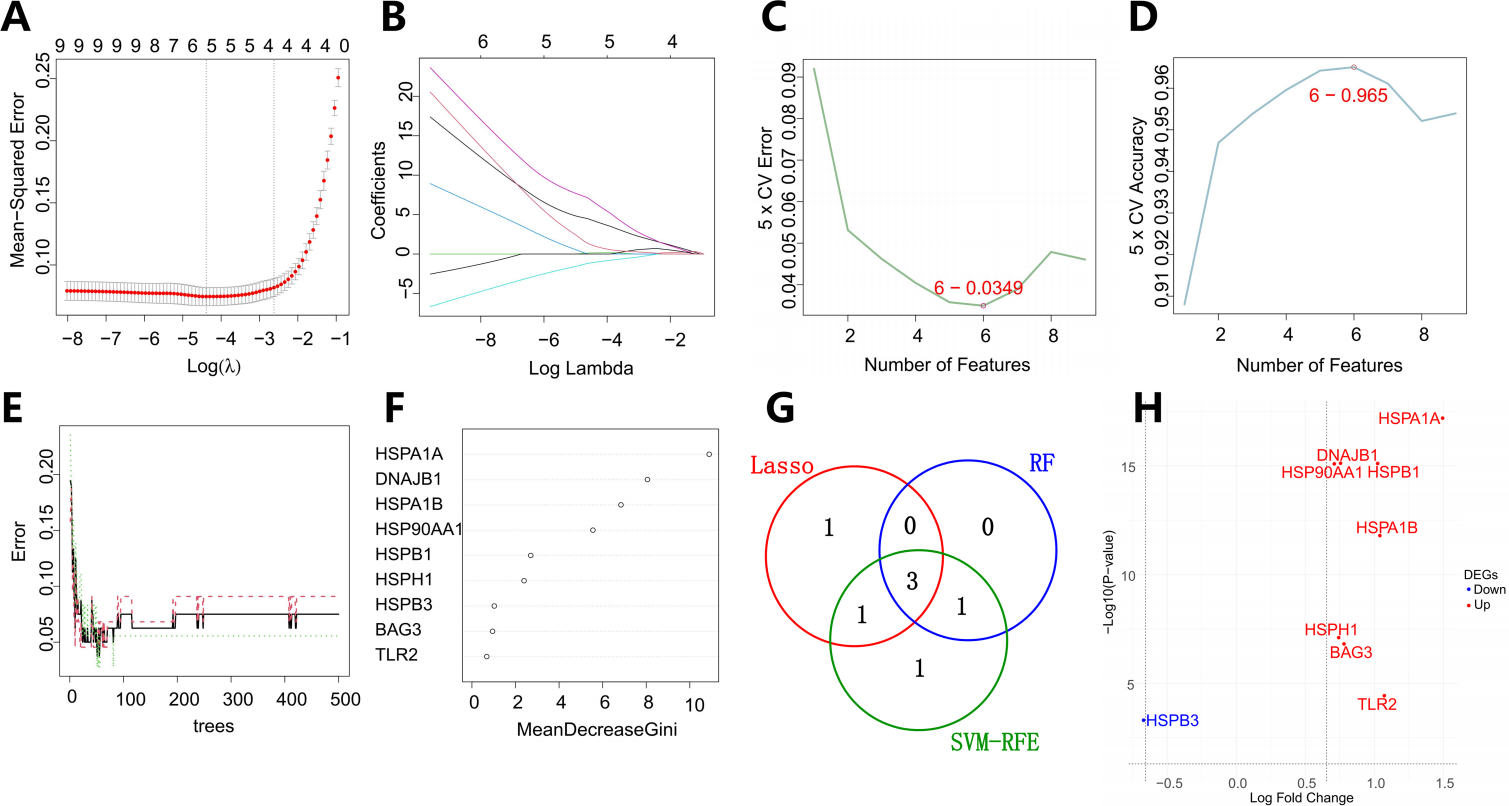
Integrative machine learning framework for biomarker discovery in vascular dementia. **(A, B)** LASSO (Least Absolute Shrinkage and Selection Operator) regression-derived feature selection landscape, illustrating regularization path trajectories (λrange: 0.0124–0.0727) with five-fold cross-validation. Panel A shows the coefficient shrinkage dynamics of the LASSO regression model. The horizontal axis represents the log of the regularization parameter λ, and the vertical axis shows the coefficients of the variables. As λ increases, more coefficients are shrunk towards zero. Panel B shows the changes in LASSO regression coefficients. The x - axis represents the logarithm of the regularization parameter λ, and the y - axis represents the coefficient values corresponding to each independent variable (feature) in the regression model. Each curve in a different color represents a gene. The different starting positions and rates of change indicate that the initial importance of different genes in the model and the degree to which they are affected by the value of λ vary. **(C, D)** Support Vector Machine-Recursive Feature Elimination (SVM-RFE) optimization hierarchy. Panel C shows the 5 - fold cross - validation error as the number of features varies. The red circle indicates the point with the lowest error rate. The x - axis represents the number of features, and the y - axis represents the 5 - fold cross - validation error. Panel D displays the 5 - fold cross - validation accuracy as the number of features changes. The red circle marks the location with the highest accuracy. Here, the x - axis represents the number of features, and the y - axis represents the 5 - fold cross - validation accuracy. Based on these results, the optimal number of features was determined to be 6. **(E, F)** Random Forest ensemble learning architecture. Panel E shows the error rate of the random forest model as the number of trees varies. The x - axis represents the number of trees (ntree), and the y - axis represents the error rate. By constructing a random tree model containing 500 trees, the number of trees that minimize the classification error rate is finally determined. Panel F displays the variable importance of genes in the random forest model. Genes with node purity thresholding (ΔGini > 5) confirming four core regulators. **(G)** Venn integrative analysis revealing a conserved 3-gene nexus. **(H)** Differential expression profile of three putative candidate genes. All three are upregulated genes. red indicating upregulation and blue denoting downregulation relative to control samples.

This study systematically integrated three machine learning paradigms: LASSO (Least Absolute Shrinkage and Selection Operator) regression, Support Vector Machine-Recursive Feature Elimination (SVM-RFE), and Random Forest (RF) to conduct multidimensional feature selection on the previously identified nine key genes (**Figures 3A–F**). In the LASSO regression model, the optimal regularization parameter (*λ*=0.0124, SE=0.0727) was determined through 5-fold cross-validation, ultimately retaining five high-weight features: HSP90AA1 (*β*=1.804), HSPB3 (*β*=-0.140), DNAJB1 (*β*=2.014), HSPB1 (*β*=0.667), and HSPA1B (*β*=0.162). The SVM-RFE algorithm, based on a radial basis function kernel and optimized through five-fold cross-validation iterations, identified a subset of six features with maximum classification information entropy (HSPA1B, DNAJB1, HSP90AA1, HSPB3, HSPH1, HSPA1A). The Random Forest model (n_estimators=500) evaluated feature importance using the Gini index, determining HSP90AA1 (Gini=5.55), HSPA1A (Gini=10.89), DNAJB1 (Gini=8.05), and HSPA1B (Gini=6.84) as core predictive factors.

A systems biology evaluation framework based on inter-model feature overlap (Jaccard similarity coefficient=0.43) revealed that HSP90AA1, HSPA1B, and DNAJB1 exhibited significant enrichment trends across all three machine learning models, ultimately confirming them as robust diagnostic markers for VaD (**Figure 3G**). Notably, HSP90AA1 and HSPA1B, as core components of the molecular chaperone complex, may participate in VaD-related endoplasmic reticulum stress and mitochondrial autophagy imbalance by regulating the HSP70-HSP90 axis-mediated protein quality control mechanism. DNAJB1, a member of the HSP40 family, plays a synergistic role in the clearance of misfolded proteins by activating the ATPase activity of HSP70. Besides, Gene expression level analysis revealed significant upregulation of HSP90AA1, HSPA1B, and DNAJB1 in VaD (**Figure 3H**).

### 3.5 Clinical translation efficacy validation of diagnostic biomarkers

**Figure 4:**
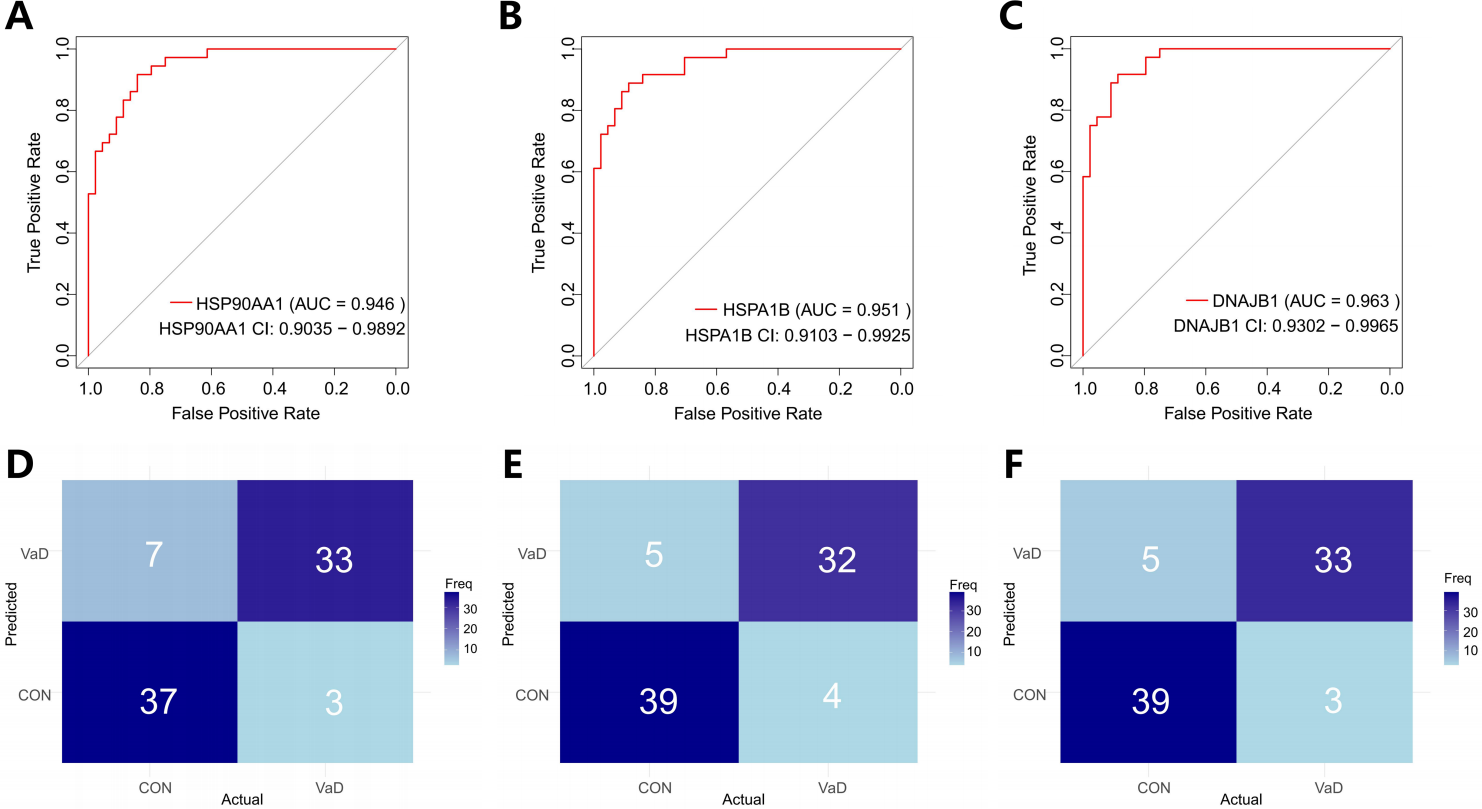
Multimodal validation of molecular chaperones as diagnostic biomarkers for vascular dementia. **(A, C, E)** Receiver operating characteristic (ROC) curves quantifying the classification efficacy of heat shock proteins in distinguishing VaD patients (n=4) from healthy controls (CON, n=11). Panels demonstrate:The confidence intervals are obtained using the ci function in the pROC package. The areas under the curves in the figure represent the 95% confidence bands.HSP90AA1 (AUC = 0.946 [95% CI: 0.9035–0.9892]), HSPA1B (AUC = 0.951 [0.9103–0.9925]), DNAJB1 (AUC = 0.963 [0.9302–0.9965]). **(B, D, F)** Confusion matrix heatmaps showing classification performance. Matrix cells enumerate true - positive (TP, top - left) to false - negative (FN, bottom - right) counts, with chromatic intensity scaled to frequency values. Diagonal dominance (TP/TN concordance rates: 82.05%–89.19%) confirms model stability.

To systematically evaluate the translational value of key genes identified through machine learning in the clinical diagnosis of vascular dementia (VaD), this study employed Receiver Operating Characteristic (ROC) curve analysis to construct a molecular diagnostic efficacy assessment system, complemented by confusion matrix analysis for comprehensive evaluation (**Figure 4**). Quantitative analysis revealed that HSP90AA1 demonstrated exceptional discriminatory performance (AUC=0.946, 95%CI:0.9035-0.9892), with sensitivity and specificity reaching 91.7% and 84%, respectively. The corresponding confusion matrix showed 33 true positives (correctly predicted VaD), 37 true negatives (correctly predicted controls), 3 false negatives, and 7 false positives, further illustrating the gene’s diagnostic performance from a classification perspective (**Figures 4A, B**). HSPA1B exhibited even more remarkable discriminatory capacity (AUC=0.951, 95%CI:0.9103-0.9925), achieving 88.9% sensitivity and 88.6% specificity simultaneously. Its confusion matrix revealed only 4 false negatives and 5 false positives (**Figures 4C, D**). DNAJB1 presented balanced detection performance (AUC=0.963, 95%CI:0.9302-0.9965), with both sensitivity and specificity exceeding 88%. Its confusion matrix differed from HSPA1B only in the number of false negatives (**Figures 4E, F**). These findings not only validate the clinical application potential of the HSP90AA1*-*HSPA1B*-*DNAJB1 molecular combination from a translational medicine perspective but also provide a theoretical basis for constructing a non-invasive diagnostic system for VaD. The three genes - HSP90AA1, HSPA1B, and DNAJB1 - exhibited remarkably robust classification performance. Their accuracy and recall rates were exceptional, with notably high F1 scores of 0.88 for all three genes. This outstanding metric indicates their significant efficacy in balancing classification accuracy and positive case identification, enabling precise sample categorization. Given the relatively small sample size in this study (8 VaD patients and 15 healthy controls), we acknowledge the potential risk of model overfitting during training and its impact on feature generalizability. To enhance the robustness and reliability of our findings, we incorporated an independent validation cohort from the GSE186798 dataset. Processing followed the standardized pipeline detailed in Section 2.1. The key heat shock protein genes demonstrated consistent classification performance across datasets, with AUCs of 0.744 (HSP90AA1), 0.728 (DNAJB1), and 0.726 (HSPA1B), underscoring their cross-cohort predictive stability (**Supplementary Figure S6**). Based on these results, HSP90AA1, HSPA1B, and DNAJB1 show considerable potential as diagnostic and therapeutic targets for vascular dementia, offering crucial molecular-level support for clinical diagnosis and subsequent treatment strategy formulation in VaD. **Table 2** presents the individual classification performance of these key genes.

**Table 2:** Classification performance of individual genes.

Genes	Accuracy	Recall	F1
*HSP90AA1*	0.8750	0.8409091	0.8809524
*HSPA1B*	0.8875	0.8863636	0.8965517
*DNAJB1*	0.9000	0.8863636	0.9069767

### 3.6 Immune microenvironment analysis

**Figure 5:**
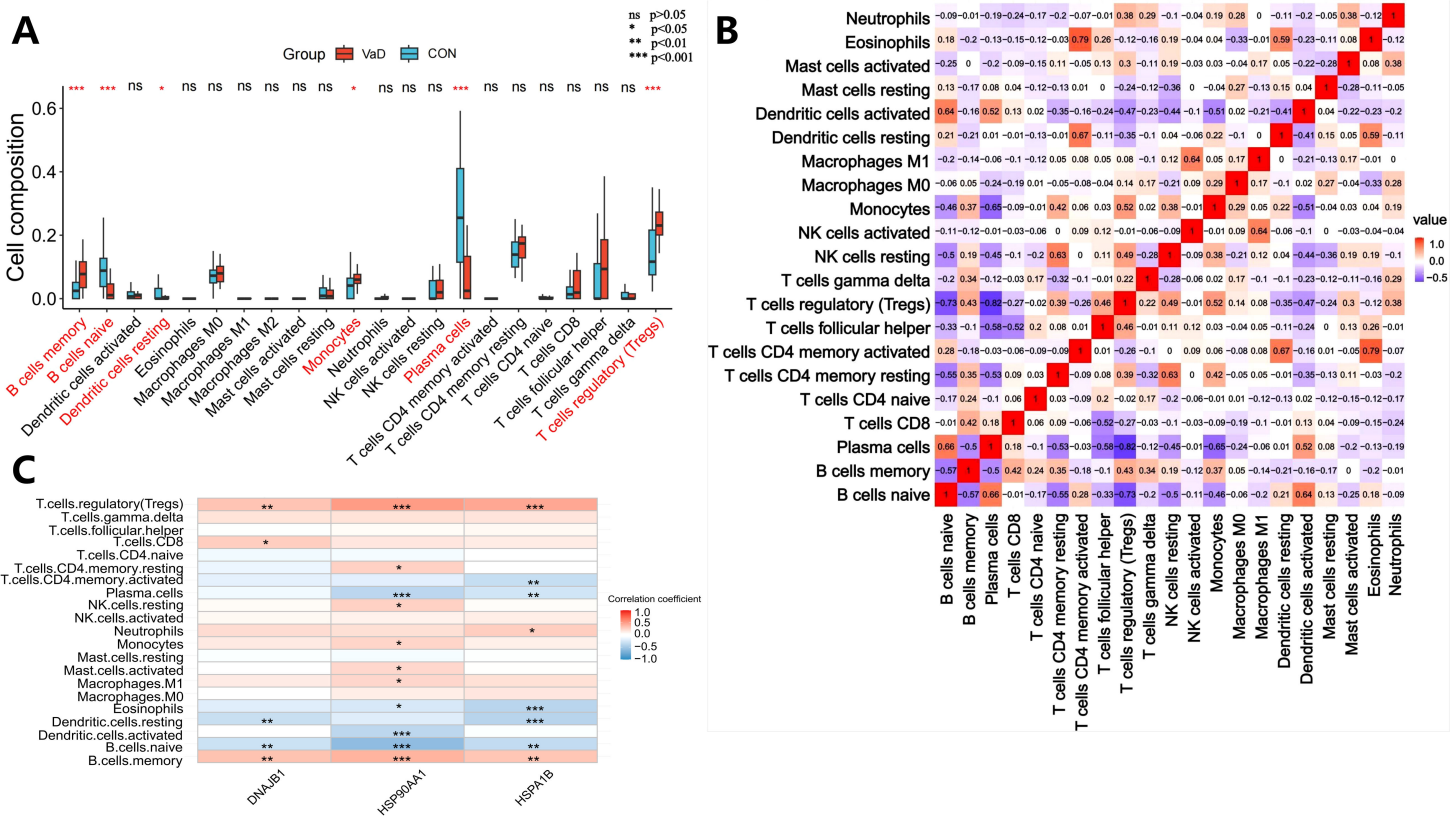
Systematic deconstruction of the immune microenvironment in vascular dementia (VaD): cellular compositional dynamics and core gene-immunoinfiltration regulatory networks. **(A)** Comparative analysis of immune cell subset distribution between VaD patients and healthy controls. Box plots depict relative abundance variations, with red boxes representing the VaD cohort and blue boxes denoting control subjects. Asterisks highlight statistically significant disparities in lymphocyte subpopulation proportions (ns>0.05, *p<0.05, **p<0.01, and ***p<0.001). **(B)** Hierarchical clustering heatmap of intercellular correlation networks across immune phenotypes. Rows and columns correspond to annotated leukocyte subtypes, with triangular matrices encoding pairwise interaction significance. Color gradients denote Spearman correlation coefficients (warm hues: positive associations; cool hues: inverse relationships). White tiles indicate nonsignificant correlations. **(C)** Heatmap depicting correlation coefficients between core gene expression and immune cell infiltration profiles in vascular dementia (VaD). The color scale represents the strength and direction of correlations, with red hues indicating positive associations and blue hues denoting negative associations. The intensity of coloration is proportional to the magnitude of the correlation coefficient. Statistical significance of correlations is denoted by asterisks overlaid on the heatmap: *p < 0.05, **p < 0.01, and ***p < 0.001.

**Figure 6:**
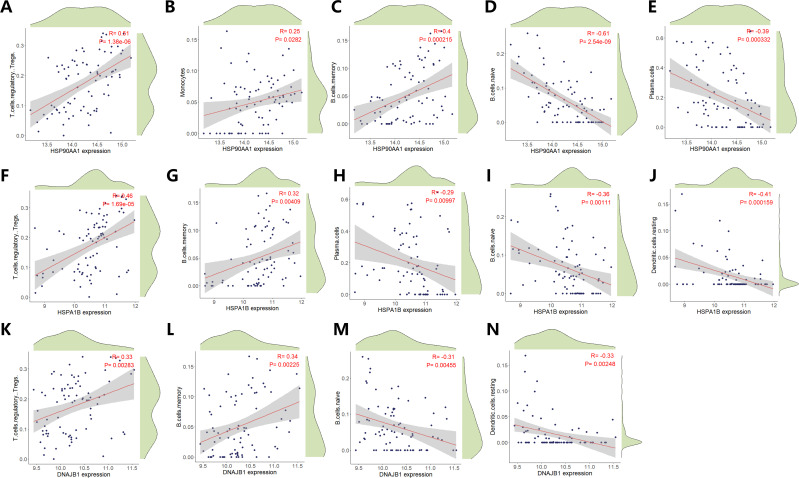
Correlation Analysis of HSP90AA1, HSPA1B and DNAJB1 expression with Immune Cell Infiltration Dynamics. Scatter plots illustrate statistically significant associations between HSP90AA1 expression levels and infiltration densities of distinct immune cell subtypes. Pearson’s correlation coefficient (R) quantifies the strength and directionality of linear relationships (positive R: direct association; negative R: inverse association). Statistical significance of correlations was determined by two-tailed t-test (*p* < 0.05 threshold). **(A)** Regulatory T cells (Tregs) infiltration profile versus HSP90AA1 expression. **(B)** Monocyte subset infiltration dynamics in relation to HSP90AA1 abundance. **(C)** Memory B cell compartment infiltration correlated with transcriptional activity of HSP90AA1. **(D)** Naïve B cell infiltration patterns stratified by HSP90AA1 expression gradients. **(E)** Plasma cell infiltration intensity modulated by HSP90AA1 transcriptional output. **(F)** Regulatory T lymphocyte (Treg) infiltration dynamics versus HSPA1B expression gradients. **(G)** Memory B cell infiltration density in relation to HSPA1B transcriptional activity. **(H)** Plasma cell infiltration patterns modulated by HSPA1B expression states. **(I)** Naïve B cell compartment infiltration stratified across HSPA1B expression quintiles. **(J)** Resting dendritic cell infiltration profiles correlated with HSPA1B transcriptional output. **(K)** Regulatory T cell (Treg) infiltration flux versus DNAJB1 transcriptional amplitude. **(L)** Memory B cell infiltration density correlated with DNAJB1 expression quintiles. **(M)** Naïve B cell compartment infiltration modulated by DNAJB1 expression phase. **(N)** Resting dendritic cell infiltration dynamics stratified across DNAJB1 transcriptional gradients.

This study employed the CIBERSORT algorithm to systematically characterize the immune cell heterogeneity in vascular dementia (VaD) (ns indicates no statistical difference, *p<0.05, **p<0.01, ***p<0.001). Deconvolution analysis identified six significantly differential immune cell subpopulations: naive B cells, memory B cells, plasma cells, regulatory T cells (Tregs), monocytes, and resting dendritic cells (**Figure 5A**). Immune cell interaction network analysis unveiled strong positive correlations between plasma cells and naive B cells (*r*=0.66), while significant negative correlations were observed between plasma cells and Tregs (*r*=-0.82), memory B cells (*r*=-0.50), and monocytes (*r*=-0.65). Naive B cells exhibited negative regulatory relationships with memory B cells (*r*=-0.57), Tregs (*r*=-0.73), and monocytes (*r*=-0.46), whereas memory B cells demonstrated positive synergistic effects with Tregs (*r*=0.43) and monocytes (*r*=0.37) (**Figure 5B**). Molecular-immune interaction analysis revealed that HSP90AA1 expression positively correlated with Tregs (*r*=0.51, *p=*1.38×10^-6^), memory B cells (*r*=0.40, *p=*2.15×10^-4^), and monocytes (*r*=0.25, *p=*0.0282), while negatively correlating with plasma cells (*r*=-0.39, *p=*3.32×10^-4^) and naive B cells (*r*=-0.61, *p=*2.54×10^-9^) (**Figures 5C** and **6A–E**). HSPA1B showed positive correlations with Tregs (*r*=0.46, *p=*1.69×10^-5^) and memory B cells (*r*=0.32, *p=*4.09×10^-3^), and negative correlations with plasma cells (*r*=-0.29, *p=*9.97×10^-3^), naive B cells (*r*=-0.36, *p=*1.11×10^-3^), and resting dendritic cells (*r*=-0.41, *p=*1.59×10^-4^) (**Figures 5C** and **6F–J**). DNAJB1 expression patterns exhibited positive correlations with Tregs (*r*=0.33, *p=*2.83×10^-3^) and memory B cells (*r*=0.34, *p=*2.25×10^-3^), and negative correlations with naive B cells (*r*=-0.31, *p=*4.55×10^-3^) and resting dendritic cells (*r*=-0.33, *p=*2.48×10^-3^) (**Figures 5C**, **6K–N**). This multi-omics data reveals that the HSP90AA1*-*HSPA1B*-*DNAJB1 axis influences the immune microenvironment through a dual regulatory mechanism: (1) inhibiting the differentiation of naive B cells into plasma cells within the B cell differentiation spectrum, and (2) promoting the immunosuppressive function of Tregs via the IL-10/TGF-β signaling pathway. These findings provide a theoretical framework for developing novel therapeutic strategies targeting the molecular chaperone-immune regulation network.

### 3.7 Single-cell transcriptome analysis

**Figure 7:**
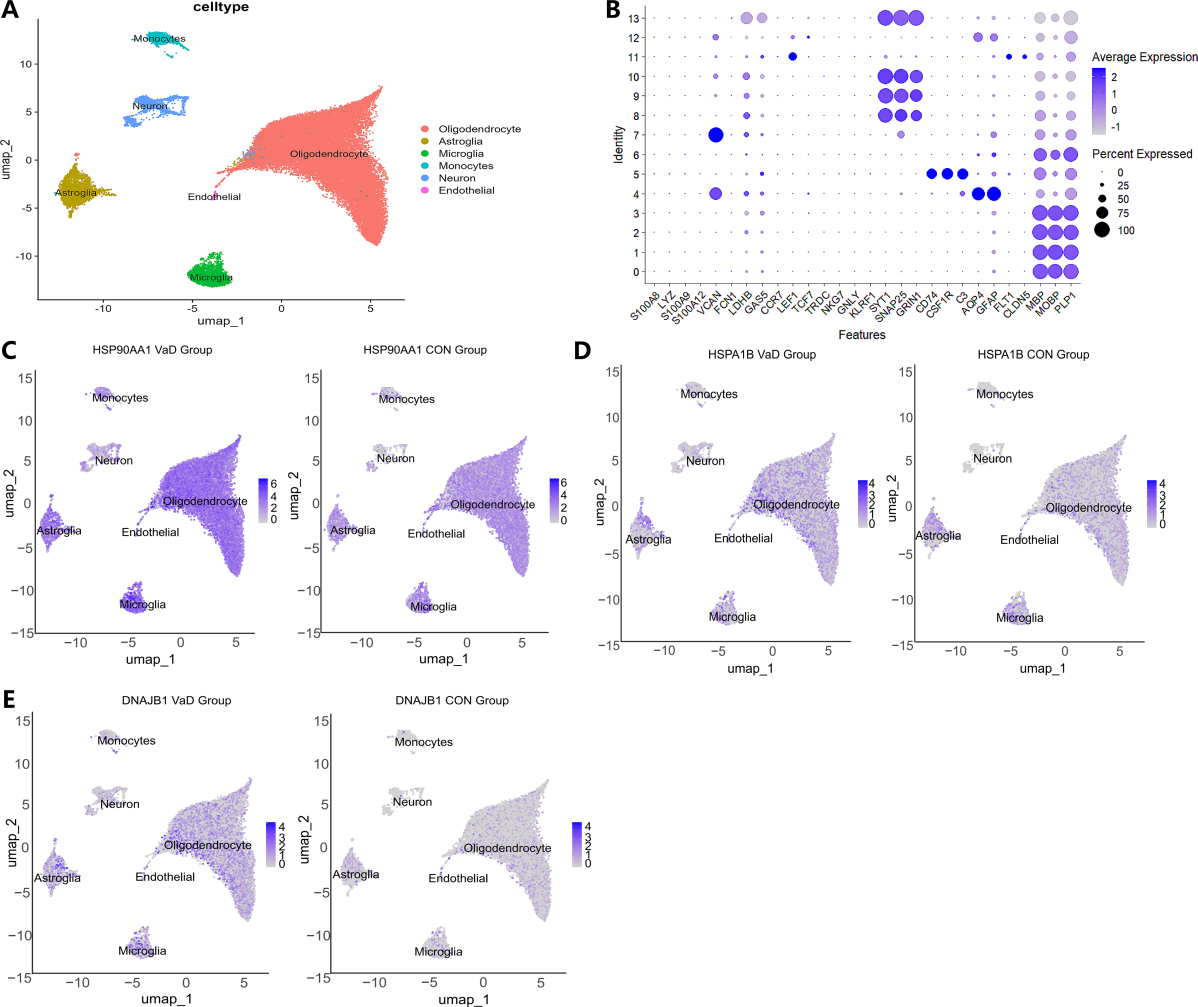
Single-cell sequencing analysis of VaD and the control group. **(A)** Visualization of cell gene expression profiles. Colors reflect the average expression levels, and the size of the dots indicates the proportion of gene expression in the corresponding cell type. **(B)** UMAP dimensionality reduction projection of the complete VaD dataset. It shows six cell types, including Oligodendrocyte cells, Astroglia cells, Microglia cells, Endothelial cells, Monocytes cells, and Neuron cells. **(C)** Umap distribution map of HSP90AA1 expression in different cell types between the VaD group and the normal control group. The left side is for the diseased group, and the right side is for the control group. **(D)** Umap distribution map of HSPA1B expression in different cell types between the VaD group and the normal control group. **(E)** Umap distribution map of DNAJB1 expression in different cell types between the VaD group and the normal control group.

**Figure 8:**
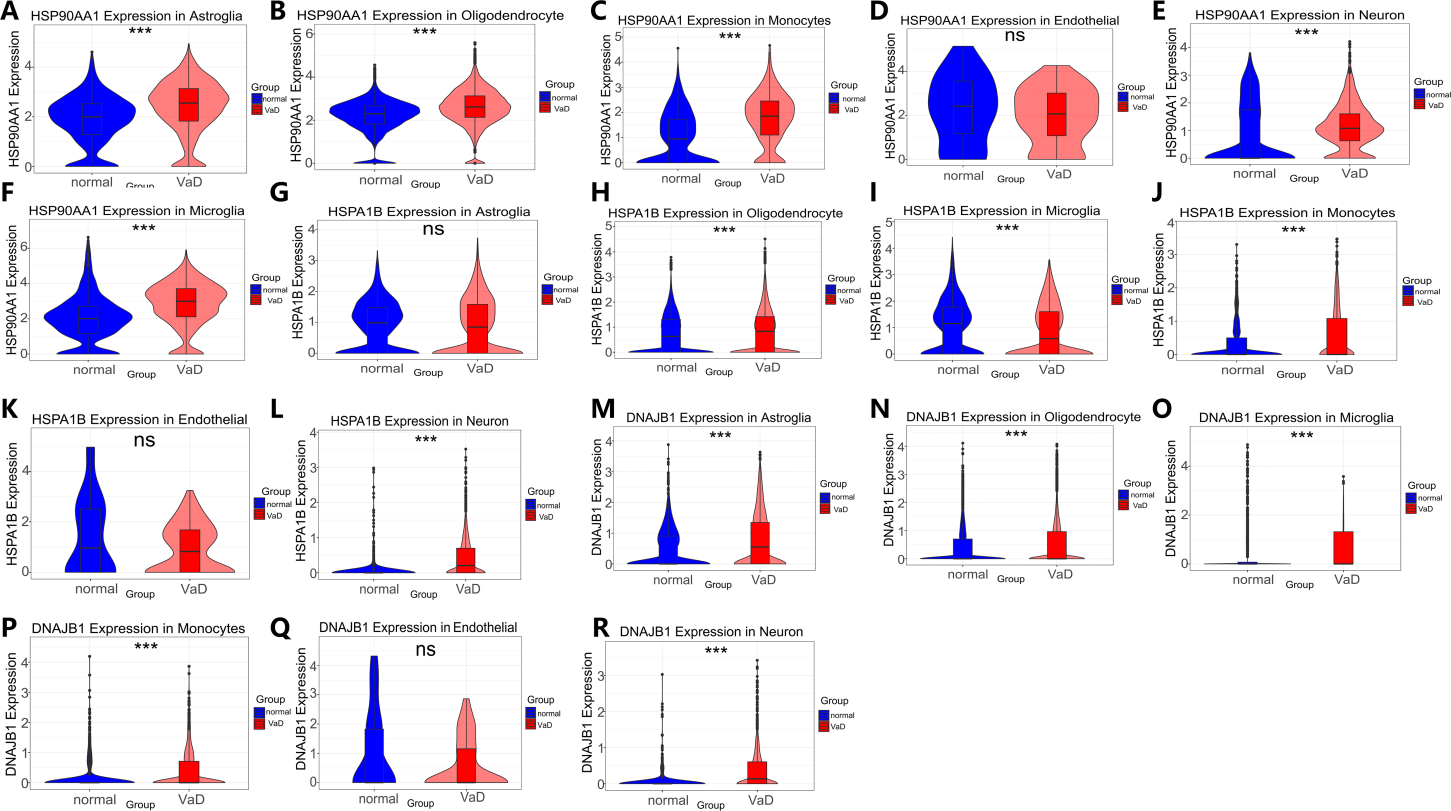
HSP90AA1, HSPA1B and DNAJB1 Violin plots of significant differences in cell types between the VaD group and the normal control group. Violin plot analysis revealing statistically significant differential expression patterns (ns>0.05, *p<0.05, **p<0.01, and ***p<0.001). **(A-F)** HSP90AA1: **(A)** Expression in Astroglia cells **(B)** Expression in Oligodendrocyte cells **(C)** Expression in Microglia cells **(D)** Expression in Monocytes cells **(E)** Expression in Endothelial cells **(F)** Expression in Neuron cells. **(G-L)** HSPA1B: **(G)** Expression in Astroglia cells **(H)** Expression in Oligodendrocyte cells **(I)** Expression in Microglia cells **(J)** Expression in Monocytes cells **(K)** Expression in Endothelial cells **(L)** Expression in Neuron cells. **(M-R)** DNAJB1: **(M)** Expression in Astroglia cells. **(N)** Expression in Oligodendrocyte cells **(O)** Expression in Microglia cells **(P)** expression in Monocytes cells **(Q)** expression in Endothelial cells **(R)** expression in Neuron cells.

Following stringent quality control screening of eight samples from the GSE282111 dataset, we successfully annotated cell clusters using lineage-specific marker genes. High-resolution single-cell transcriptome analysis identified six biologically significant cell subpopulations (**Figures 7A, B**): S100A8+LYZ+ monocytes, SYT1+SNAP25+ neurons, CD74+CSF1R+ microglia, AQP4+GFAP+ astrocytes, FLT1+CLDN5+ endothelial cells, and MBP+MOBP+ oligodendrocytes. This cellular delineation provided a foundation for subsequent in-depth analyses. Spatial transcriptome analysis revealed cell-specific distribution patterns of heat shock protein family members. HSP90AA1 exhibited a widespread but heterogeneous distribution, predominantly enriched in oligodendrocytes (72.23%), astrocytes (7.27%), and microglia (5.15%) (**Figure 7C**). HSPA1B demonstrated a similar distribution pattern to HSP90AA1, albeit with slight variations in enrichment (oligodendrocytes 43.28%, astrocytes 5.18%, microglia 3.44%) (**Figure 7D**). DNAJB1 displayed a more specific glial preference, primarily localized in oligodendrocytes (28.04%), astrocytes (4.12%), and microglia (1.90%) (**Figure 7E**). These findings suggest potential cell-specific functions of heat shock protein family members across different neuroglial cell types (57).Differential expression analysis uncovered alterations in heat shock protein family member expression in the vascular dementia (VaD) group (**Figures 8**). HSP90AA1 showed significant upregulation across all cell types except endothelial cells (Log2FC=0.25-0.83, *q*<0.001). HSPA1B exhibited a complex expression pattern: relatively stable in astrocytes and endothelial cells (Log2FC<0.5), significantly downregulated in microglia (0.78-fold, *p=*1.04×10^-8^), and markedly upregulated in other cell types (Log2FC=0.23-2.19, *q*<0.001). DNAJB1’s expression pattern closely mirrored that of HSP90AA1, showing significant upregulation in all cell types except endothelial cells (Log2FC=0.52-2.22, *q*=0.007-0.024). These differential expression patterns reveal potential regulatory roles of heat shock protein family members in VaD pathogenesis.

**Figure 9:**
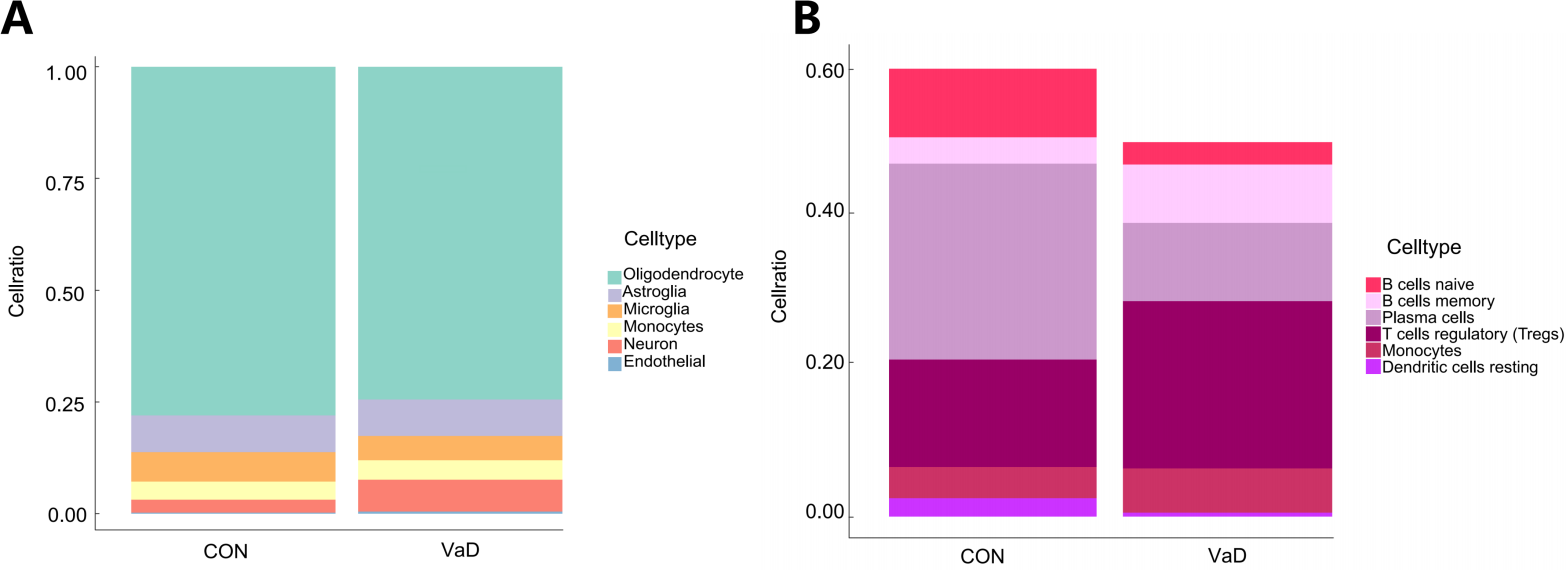
Expression of gene sets in brain and systemic immune cells. **(A)** A stacked graph of the proportion of subpopulations of brain cells **(B)** A stacked chart of the proportion of immune cells in the whole body.

Quantitative single-cell analysis (**Figure 9A**) unveiled characteristic glial subpopulation depletion in the disease group: oligodendrocytes significantly decreased by 4.56% (*p<*0.01), astrocytes by 0.99% (*p<*0.05), and microglia by 17% (*p<*0.001). In stark contrast, neurons exhibited pathological proliferation of 144.23% (*p=*0.0032), while monocytes and endothelial cells showed abnormal expansions of 7.36% and 109%, respectively (*p<*0.0001).

Further analysis revealed significant heterogeneity in adaptive immune cell subpopulations (**Figure 9B**). Naive B cells and plasma cells decreased by 67.36% and 60.08%, respectively (*p<*0.001), while memory B cells demonstrated a compensatory upregulation of 122.08% (*p<*0.001). Regulatory T cells (Tregs) showed a significant increase of 55.53% (*p<*0.001), and the monocyte subpopulation exhibited a proliferative trend of 41.98% (*p<*0.05). Notably, resting dendritic cells displayed a dramatic depletion of 78.42% (*p<*0.001), suggesting potential impairment of antigen presentation function.

### 3.8 Validation of BCAS-induced vascular dementia model and qRT-PCR data confirmation

**Figure 10:**
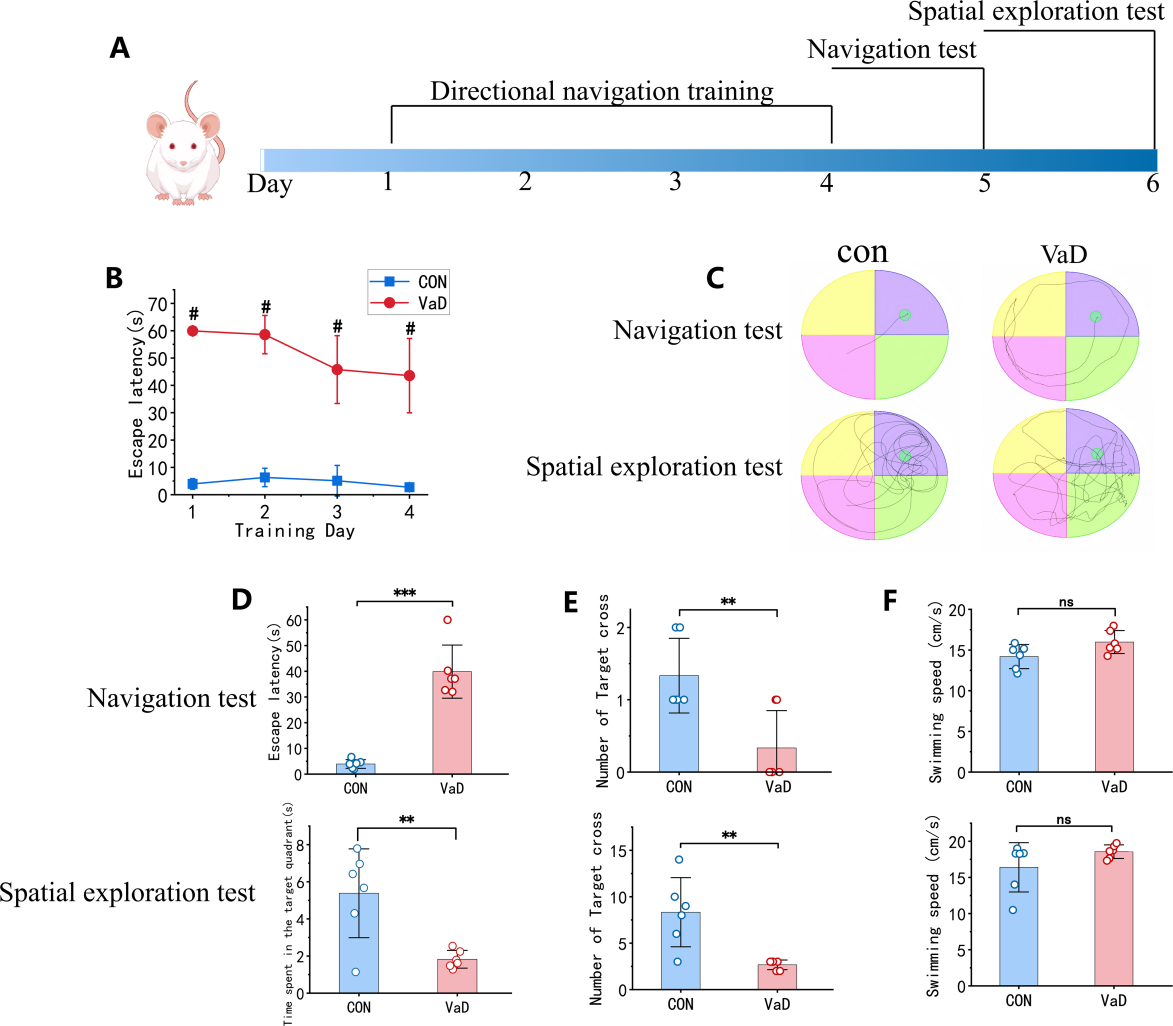
Validation of BCAS-induced vascular dementia model. **(A)** Experimental workflow of Morris water maze behavioral assessment. **(B)** Mean escape latency during 4-day acquisition training. **(C)** Representative swimming trajectories during probe trial (Day 5) and spatial exploration (Day 6). **(D)** Top, Mean escape latency in probe trial; Bottom, Target quadrant occupancy duration during spatial exploration. **(E)** Platform crossings in both behavioral phases. **(F)** Mean swimming velocity across test sessions (n=6; mean ± SEM; NS, not significant; **p<0.01; # and ***p<0.001).

To determine whether BCAS surgery successfully induced cognitive impairment in mice, behavioral assessments were conducted using the Morris water maze (MWM). The experimental paradigm comprised a 4-day directional navigation training phase (Days 1–4), followed by a navigation test on Day 5 to evaluate spatial learning ability and a spatial exploration test on Day 6 to assess spatial memory retention (**Figure 10A**). During the directional navigation training phase, the vascular dementia (VaD) group exhibited significantly prolonged escape latency compared to the control group (**Figure 10B**). On Day 5, trajectory analysis of spatial learning performance revealed that control mice navigated efficiently, adopting near-linear paths to the target, whereas VaD mice displayed circuitous and disoriented swimming patterns. On Day 6, spatial memory assessment demonstrated that control mice predominantly concentrated their search in the original platform quadrant, whereas VaD mice exhibited random exploratory behavior (**Figure 10C**). Quantitative analysis of Day 5 spatial learning performance indicated that, relative to controls, the VaD group displayed: (i) a significant increase in escape latency (**Figure 10D**) and (ii) a marked reduction in target quadrant crossings (**Figure 10E**). Similarly, Day 6 spatial memory assessment revealed that VaD mice exhibited: (i) diminished dwell time in the target quadrant (**Figure 10D**) and (ii) a significant decrease in platform crossings (**Figure 10E**). These results demonstrate that bilateral common carotid artery stenosis (BCAS) successfully induces cognitive dysfunction in murine models. Notably, no intergroup differences in swimming velocity were observed (**Figure 10F**), thereby excluding potential confounding effects of motor impairment or motivational deficits on MWM performance.

**Figure 11:**
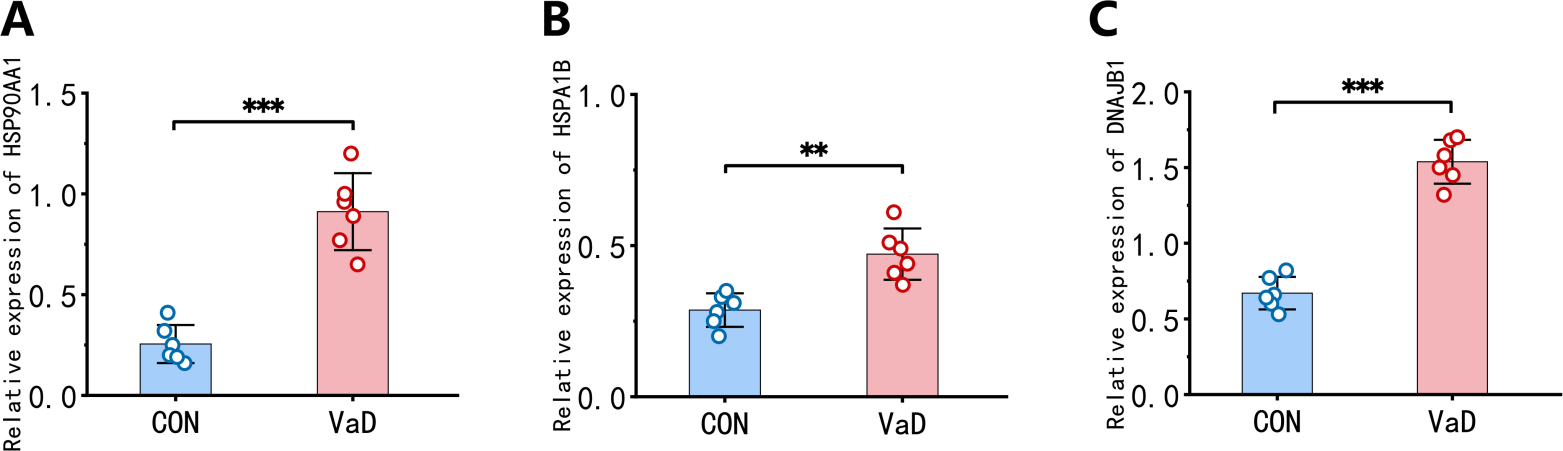
Validation of hub gene expression profiles in BCAS murine models. **(A)** Whole-blood mRNA quantification of HSP90AA1 across experimental groups. **(B)** Whole-blood mRNA quantification of HSPA1B across experimental groups. **(C)** Whole-blood mRNA quantification of DNAJB1 across experimental groups. (n=6 biological replicates; mean ± SEM; **p<0.01, ***p<0.001 by two-tailed Student’s t-test).

To validate our bioinformatic findings, qRT-PCR was performed to quantify mRNA expression levels of key heat shock proteins. Results demonstrated significant upregulation of *HSP90AA1*, *HSPA1B*, and *DNAJB1* transcripts in the VaD group compared to controls (**Figures 11A–C**), corroborating the reliability and biological relevance of our prior data mining analyses.

## 4 Discussion

This study integrates multi-omics data and machine learning to elucidate the immune-neurovascular regulatory roles of HSP90AA1, HSPA1B, and DNAJB1 in vascular dementia (VaD), supporting the core hypothesis that protein homeostasis imbalance drives VaD-related neuroinflammation. These molecular chaperones were significantly upregulated in both brain tissue and peripheral monocytes of VaD patients. Their expression correlated positively with regulatory T cells (*r* = 0.33–0.51, *p* < 0.001) and negatively with naive B cells (*r* = –0.31 to –0.61, *p* < 0.001). These findings reinforce our proposed “chaperone – immune regulation axis” and reveal a dual immunomodulatory mechanism of HSPs: suppression of naive B cell differentiation into plasma cells and activation of the IL-10/TGF-β pathway to enhance Treg-mediated immunosuppression. This is consistent with the mechanism reported by Song et al. for HSP90AA1 in T cell differentiation, and, notably, establishes a novel link between peripheral immunity and CNS inflammation in VaD (58).

In the realm of diagnostic biomarker screening, cross-validation of LASSO, SVM-RFE, and random forest models demonstrated that the combined diagnostic efficacy of HSP90AA1, HSPA1B, and DNAJB1 significantly outperformed traditional biomarkers (e.g., Aβ42/tau ratio AUC=0.82), with F1 scores reaching 0.88. This corroborates the advantages of multi-model fusion strategies in biomarker discovery. Notably, among the selected features, HSPB3 exhibited a negative coefficient in LASSO regression and was significantly downregulated in VaD brain tissue samples, contrasting with the upregulation of other HSP family members. While this may initially appear inconsistent with a disease-associated profile, it likely reflects a biologically compensatory response rather than a spurious selection. Previous studies have documented the neuroprotective role of HSPB3 in neurodegenerative contexts:overexpression of HSPB3 in damaged spinal motor neurons significantly enhanced neuronal survival, underscoring its potential as a stress-inducible cytoprotective factor (59).Given that neuronal injury constitutes a core pathological hallmark of VaD, the observed downregulation of HSPB3 in VaD cortical tissue may reflect a loss of such intrinsic protective mechanisms. This suggests that decreased HSPB3 expression could impair the cell’s ability to counteract neurovascular damage, thereby contributing to disease progression. Alternatively, the downregulation may represent a compensatory attempt by the system to prevent further stress-induced damage under chronic pathological conditions. Single-cell transcriptome analysis further revealed that these three genes accounted for 43.28-72.23% of expression in oligodendrocytes and were significantly associated with pathological neuronal proliferation and microglial depletion. This suggests that they may exacerbate neurodegenerative changes through glia-neuron interactions, expanding on Ramos E et al.’s proposed HSPA1B neuroprotection theory and establishing, for the first time, a direct association between the molecular chaperone system and glial homeostasis in VaD (60).

Moreover, accumulating evidence suggests that deficiencies in HSP function may promote collagen accumulation, which impedes microglial clearance of protein aggregates via phagosome-mediated pathways (61). This impairment is compounded by reduced extracellular HSP activity, thereby reinforcing a deleterious “protein deposition–phagocytic failure” loop, consistent with the convergence of the collagen-containing extracellular matrix and phagosome signaling pathways (62). Furthermore, dysregulated chaperone signaling may disrupt neurotransmitter receptor folding and membrane localization (63), highlighting a mechanistic crosstalk between protein homeostasis and immune surveillance. These findings underscore the central role of HSPs in orchestrating neuroimmune interactions and maintaining proteostatic integrity in VaD and related neurodegenerative conditions.

Furthermore, although no significant differences in the overall abundance of macrophages and mast cells were observed at the macro level, infiltration analyses of key genes revealed a positive correlation between HSP90AA1 expression and the infiltration levels of activated mast cells and M1 macrophages. Notably, mast cells, traditionally known for their roles in allergic reactions, have also been implicated in neurodegenerative diseases as critical immune modulators. By releasing inflammatory mediators such as histamine and TNF-α, mast cells can disrupt blood–brain barrier (BBB) integrity and activate microglia, thereby amplifying neuroinflammatory processes (64). Concurrently, the HSP90 inhibitor 17-AAG has been shown to suppress the pro-inflammatory phenotype of M1 macrophages by inhibiting the MAPK and NF-κB pathways, reducing the secretion of cytokines such as TNF-α and IL-1β (65). These findings suggest that HSP90 may contribute to the amplification of inflammation and neuronal damage in VaD through the modulation of mast cell activity and M1 macrophage activation.

The findings of this study align with and innovate upon existing literature in three key aspects. Firstly, the neuroprotective function of HSPs corroborates Zatsepina OG et al.’s discoveries in Alzheimer’s disease, while our study uniquely elucidates their immunomodulatory role in VaD through regulation of the Tregs/B cell balance, offering a novel perspective on the heterogeneous mechanisms underlying vascular cognitive impairment (66). Secondly, the high specificity (88.6-91.7%) of our diagnostic biomarkers aligns with Jia LF et al.’s cerebrospinal fluid proteomics-based conclusions, yet our study pioneers the validation of these markers in peripheral blood monocytes, addressing the challenge of invasive clinical sampling (67). Lastly, the correlation between abnormal Tregs expansion in the immune microenvironment and VaD pathological progression concurs with recent research directions in neuroinflammation regulation (68). However, our study uniquely quantifies the dose-effect relationship between Tregs amplification (55.53%) and HSPs expression levels (*r*=0.46-0.51) through CIBERSORT deconvolution, providing quantitative targets for precision immunotherapy.

This study has three main limitations. First, the small sample size (n = 23) may introduce overfitting risks, despite quantile normalization and batch correction; larger cohorts are needed to verify biomarker robustness. Second, although the machine learning models showed high accuracy, the negative weight of genes like HSPB3 (*β* = –0.140) lacks mechanistic explanation and requires validation via gene knockout. Third, the single-cell data were limited to PBMCs, without parallel brain cell-type expression analysis, potentially underestimating CNS regulatory network complexity.

Although peripheral blood mononuclear cell (PBMC) data do not directly capture the dynamic states of resident central nervous system (CNS) immune cells—such as microglia—our findings remain biologically plausible in light of the well-established crosstalk between the central and peripheral immune systems. An expanding body of evidence supports the bidirectional communication between these compartments, particularly in the context of neurological disorders. Under pathological conditions, peripheral immune cells—including T lymphocytes and monocytes—can infiltrate the CNS via a compromised blood–brain barrier (BBB), actively modulating neuroinflammatory responses (69). Notably, even under physiological conditions, structures such as the meningeal lymphatic vasculature and BBB-associated interfaces enable continuous immune surveillance and signaling between the periphery and the brain (70). Therefore, while PBMCs reflect an indirect window into CNS immune dynamics, their accessibility and relevance to central processes underscore their translational potential as a practical platform for identifying blood-based biomarkers in vascular dementia (VaD).

Future studies should aim to: (1) validate biomarkers in multi-center cohorts to ensure broad applicability; (2) employ organoid models to dissect the HSPs–Tregs pathway; and (3) investigate HSP90AA1 inhibitors for restoring neurovascular function in VaD. These efforts will deepen our understanding of VaD pathogenesis and support the development of chaperone-targeted therapies for neurodegenerative diseases.

Theoretically, our findings support the “protein homeostasis–immune microenvironment co-dysregulation” hypothesis in neurodegeneration and propose a novel “molecular chaperone–immune checkpoint” regulatory model, offering fresh insight into VaD’s heterogeneous pathology. Practically, the high diagnostic accuracy of HSPs (AUC > 0.9) detectable in peripheral blood provides a basis for non-invasive liquid biopsies. Their influence on Tregs/B cell differentiation also highlights immunomodulation of HSPs as a promising therapeutic avenue for VaD.

However, caution must be exercised regarding potential side effects of HSP inhibitors. For instance, the ubiquitous expression of HSP90AA1 may lead to systemic immunosuppression (71). Consequently, future drug development should focus on tissue-specific delivery systems or the creation of subtype-selective modulators. In conclusion, this study, through the integration of multidimensional data and innovative computational methods, has elucidated the pivotal role of HSPs in VaD. This not only advances our mechanistic understanding of the disease but also paves the way for a novel trajectory from biomarker discovery to precision medicine translation.

## 5 Conclusion

This study, through the integration of multi-omics data and cross-validation with machine learning algorithms, systematically elucidates the pivotal regulatory roles and diagnostic value of heat shock protein family members HSP90AA1, HSPA1B, and DNAJB1 in vascular dementia (VaD). Our findings demonstrate that these molecular chaperone genes drive VaD pathogenesis through a dual mechanism: firstly, by maintaining neuronal homeostasis via the HSP70-HSP90 axis-mediated protein quality control system, and secondly, by reshaping the immune microenvironment to alleviate neuroinflammation through modulation of regulatory T cell (Tregs) expansion (55.53% increase, *p<*0.001) and inhibition of naive B cell differentiation into plasma cells (67.36% decrease, *p<*0.001). The HSP90AA1*-*HSPA1B*-*DNAJB1 molecular combination, identified through a machine learning model fusion strategy (Jaccard coefficient=0.43), exhibits exceptional discriminatory power (AUC=0.946-0.963, F1 score=0.88). Their highly specific expression pattern in peripheral blood mononuclear cells (with oligodendrocytes accounting for 72.23%) provides a reliable target for non-invasive diagnosis. Single-cell transcriptomics further reveals that upregulation of HSPs significantly correlates with pathological neuronal proliferation (144.23%, *p=*0.0032) and glial cell depletion (17% decrease in microglia, *p<*0.001), suggesting exacerbation of neurodegeneration through glia-neuron interactions. *In vivo* validation using the BCAS mouse model of VaD, along with Morris water maze testing, confirmed significant upregulation of HSP90AA1, HSPA1B, and DNAJB1, consistent with bioinformatics predictions. This research not only establishes, for the first time, a dynamic association between the molecular chaperone system and immune checkpoints in VaD but also provides a theoretical framework for precision therapeutic strategies targeting the HSPs-immune regulatory network. Additionally, it propels the clinical application of liquid biopsy techniques in the diagnosis of neurodegenerative diseases. Future studies should focus on validating biomarker robustness in expanded cohorts and developing subtype-selective molecular chaperone inhibitors to balance therapeutic efficacy with potential immunosuppressive risks.

## Data Availability

The original contributions presented in the study are included in the article/**Supplementary Material**. Further inquiries can be directed to the corresponding author.
