# Supplementary material for: Multi-omics exploration of chaperone-mediated immune-proteostasis crosstalk in vascular dementia and identification of diagnostic biomarkers
Source: Front Immunol. 2025 Jul 30;16:1615540. doi: 10.3389/fimmu.2025.1615540 (PMC12343264; doi:10.3389/fimmu.2025.1615540)
Supplement: Supplementary file 6 [file Supplementaryfile1.docx]

Supplementary Material

# Supplementary Data

## Supplementary Figures


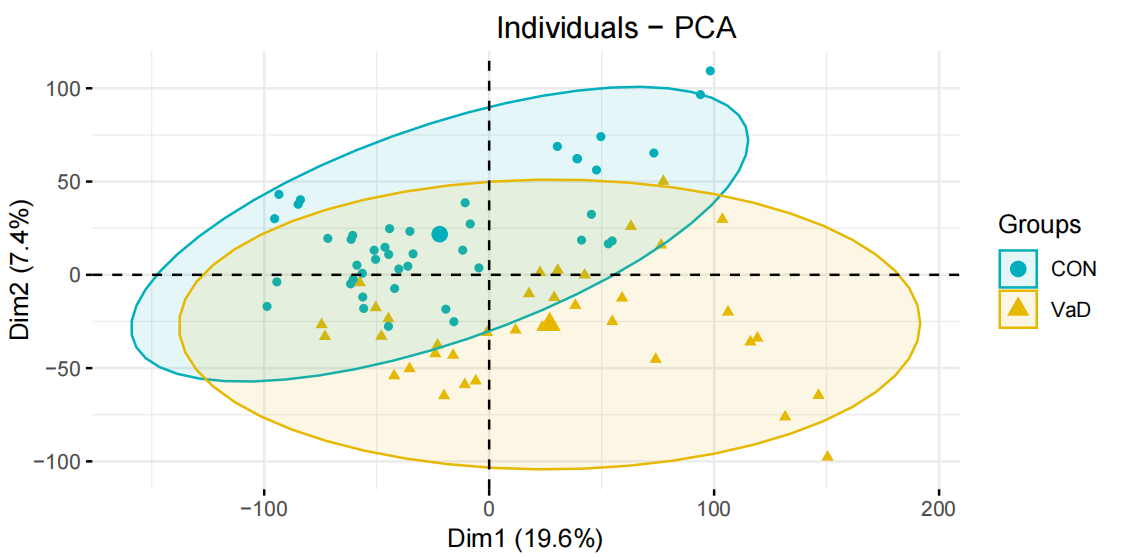


**Supplementary Figure 1.** Principal Component Analysis (PCA) of the main components for patients with vascular dementia and normal control samples. involving a total of 15 samples (11 from the normal group and 4 from the vascular dementia group). The data were standardized before PCA analysis. This figure illustrates the distribution of samples in the principal component analysis (PCA) reduced dimension space between the normal control group (blue points) and the vascular dementia group (red points).


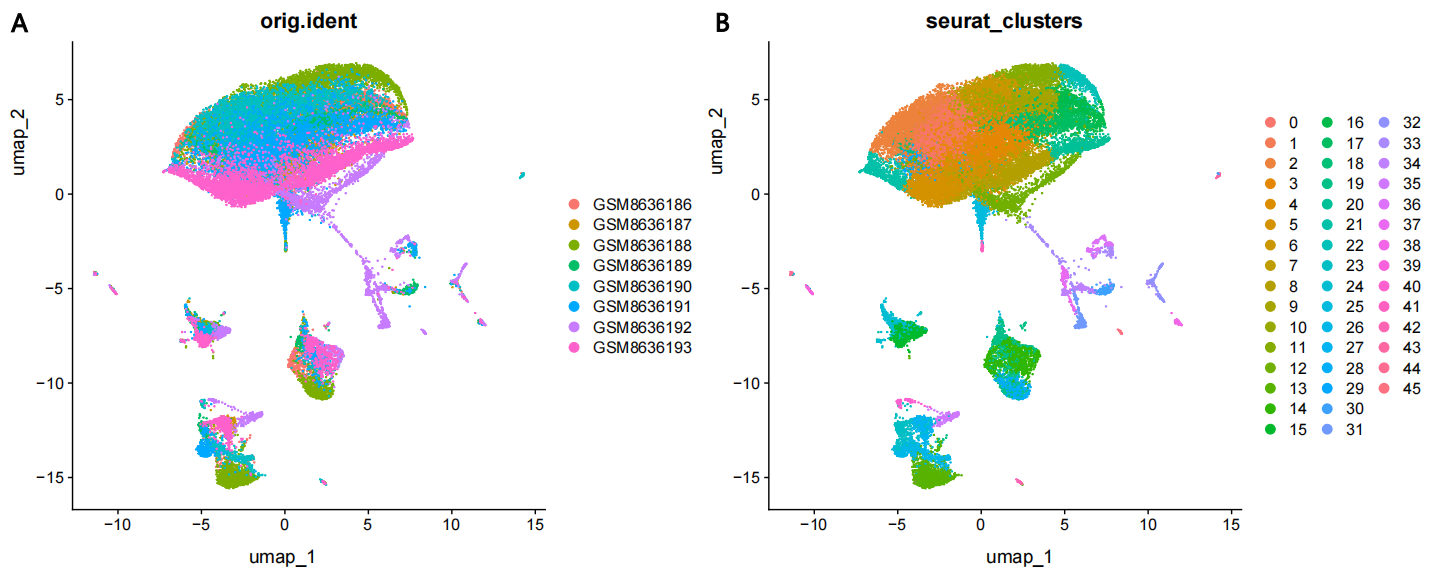


**Supplementary Figure 2.** The UMAP (Uniform Manifold Approximation and Projection, Uniform Manifold Approximation and Projection) plot of the raw data. (A) A single-cell data UMAP visualization based on the original data identifiers (orig.ident). Different colors represent different original datasets (GSM8636186-GSM8636193), showing the distribution of cells from each original sample source in the UMAP two-dimensional space (umap_1 and umap_2). (B) A single-cell data UMAP visualization based on the Seurat clustering results (seurat_clusters). Different colors correspond to different clusters (numbered 0-45), illustrating the distribution of cell populations clustered based on gene expression characteristics in the UMAP two-dimensional space (umapd_1 and umapd_2). Cells with the same color exhibit similar gene expression patterns.


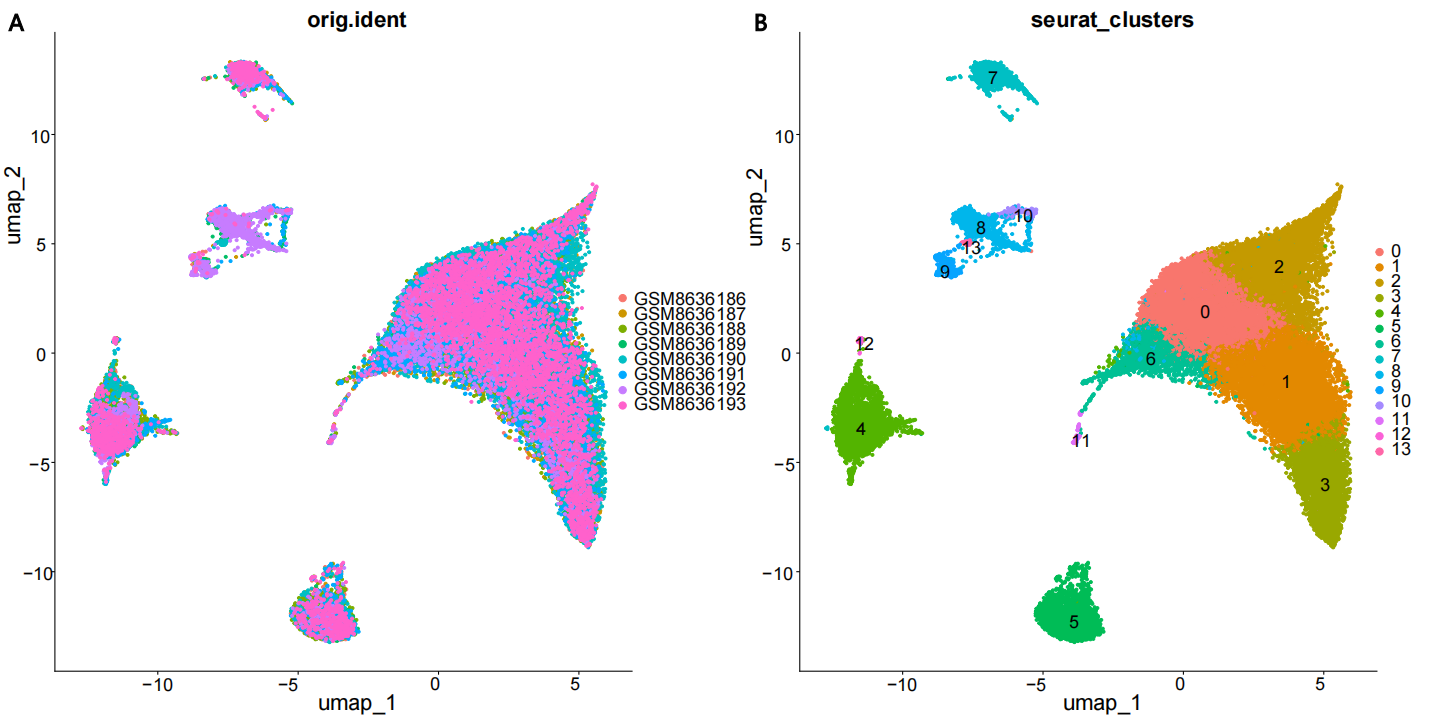


**Supplementary Figure 3.** The UMAP (Uniform Manifold Approximation and Projection) plot of the data after CCA batch effect correction. (A) UMAP visualization of single-cell data, with different colors representing different original datasets (such as GSM8636186-GSM8636193). (B) UMAP visualization of single-cell data based on Seurat clustering results (seurat_clusters), with different colors representing different clusters (numbered 0-13).


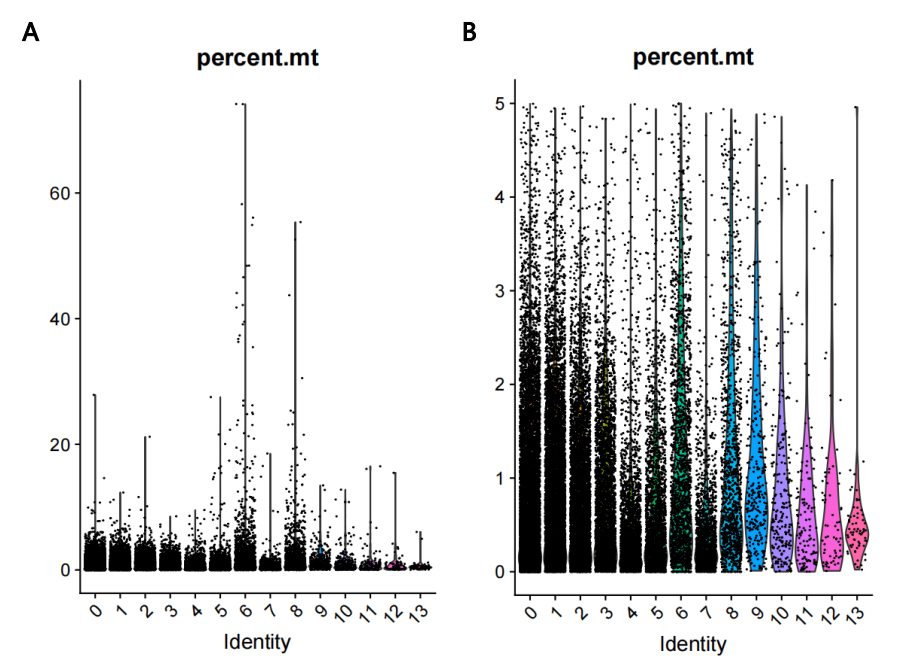


**Supplementary Figure 4.** Figure of mitochondrial gene expression ratio quality control results. (A) Original data on mitochondrial gene expression ratios, where the vertical axis represents the numerical values of mitochondrial gene expression ratios, and the horizontal axis indicates different cell clusters of 13, with significant differences in mitochondrial gene expression ratios between groups. (B) Data after quality control on mitochondrial gene expression ratios, where cells with percent.mt greater than 5% were filtered out, concentrating the overall mitochondrial gene expression ratios at a lower level (0-5%), and the distribution among different groups also changed.


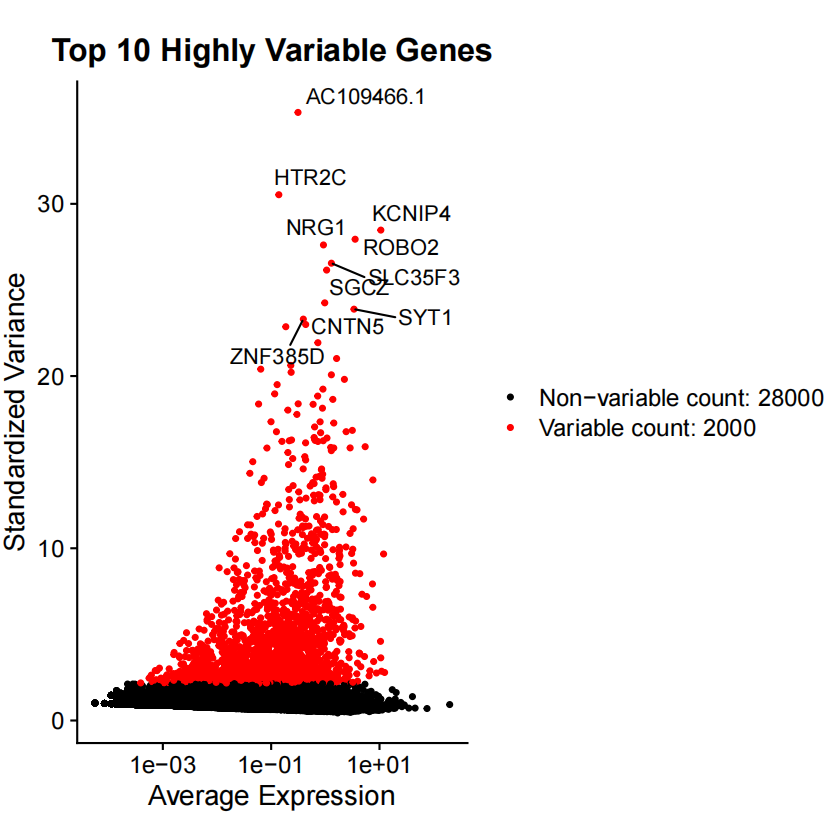


**Supplementary Figure 5.** Top 10 Visualization of highly variable genes. The horizontal coordinate represents the average expression of genes, and the vertical coordinate represents the standardized variance of genes. Black points: represent non-highly variable genes, the number is 28000, and red points: represent highly variable genes, the number is 2000.


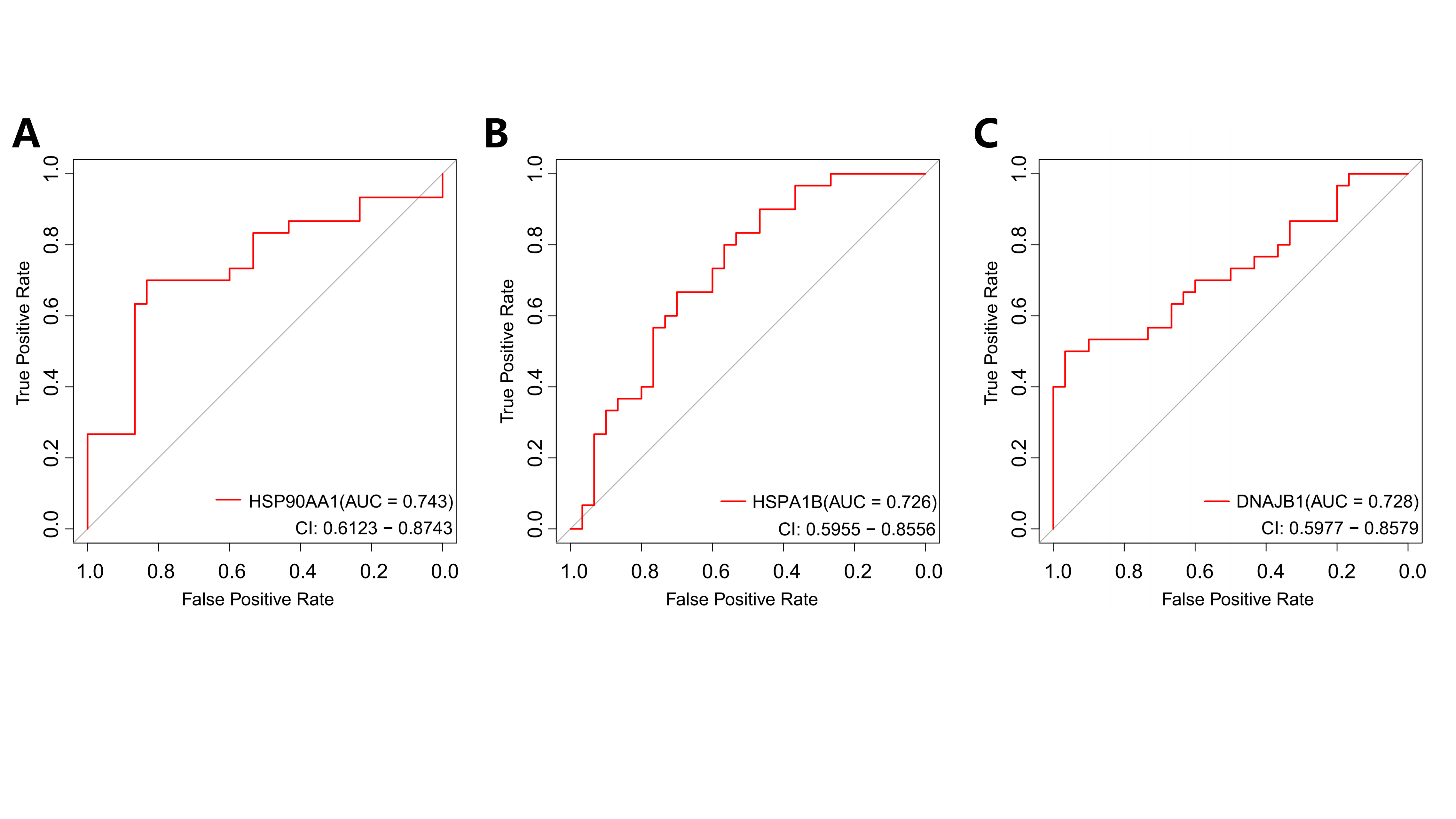


**Supplementary Figure 6.** ROC Curves of Molecular Chaperone Genes (External Validation Cohort). Receiver operating characteristic (ROC) curves were used to evaluate the classification performance of key heat shock proteins in distinguishing vascular dementia (VaD, n = 30) from healthy controls (CON, n = 30). The 95% confidence intervals were calculated using the ci function of the pROC package. The area under the curve (AUC) values for each gene were as follows: HSP90AA1 (AUC = 0.743, 95% CI: 0.6123–0.8743), HSPA1B (AUC = 0.726, 95% CI: 0.5955–0.8556), and DNAJB1 (AUC = 0.728, 95% CI: 0.5977–0.8579).

## Supplementary Tables

**Supplementary Table 1.** three-tier Gene Ontology (GO) annotation ----Biological Process (BP).

**Supplementary Table 2.** three-tier Gene Ontology (GO) annotation ----Molecular Function (MF) .

**Supplementary Table 3.** three-tier Gene Ontology (GO) annotation ----and Cellular Component (CC).

**Supplementary Table 4.** the Kyoto Encyclopedia of Genes and Genomes (KEGG) pathway data.

**Supplementary Table 5.** Data on the proportion of expression of samples in cell subpopulations.
